# An enhancing diagnostic pulmonary diseases diagnostic method for differentiating talaromycosis from tuberculosis

**DOI:** 10.1016/j.isci.2025.111867

**Published:** 2025-01-22

**Authors:** Ying Zhou, Pengchen Lin, Lijing Xia, Ali Asghar Heidari, Yi Chen, Lei Liu, Huiling Chen, Chengye Li, Yuping Li

**Affiliations:** 1 Department of Pulmonary and Critical Care Medicine, The First Affiliated Hospital of Wenzhou Medical University, Wenzhou 325000, China; 2 School of Surveying and Geospatial Engineering, College of Engineering, University of Tehran, Tehran, Iran; 3 Key Laboratory of Intelligent Informatics for Safety & Emergency of Zhejiang Province, Wenzhou University, Wenzhou 325035, China; 4 College of Computer Science, Sichuan University, Chengdu, Sichuan 610065, China; 5 College of Computer Science and Artificial Intelligence, Wenzhou University, Wenzhou 325035, P.R. China

**Keywords:** Public health, Machine learning

## Abstract

Talaromycosis (TSM) affects immunocompromised individuals, particularly those with human immunodeficiency virus (HIV)/acquired immunodeficiency syndrome (AIDS), causing varied pulmonary abnormalities on chest computed tomography (CT). These features overlap with pulmonary tuberculosis, making accurate differentiation essential for appropriate treatment. This study utilized real patient data from the First Affiliated Hospital of Wenzhou Medical University. A machine learning model, termed bIPCACO-FKNN, was developed, integrating an ant colony optimization (ACO) algorithm with a fuzzy k-nearest neighbors (FKNNs) classifier. This model introduces an incremental proportional-integral-derivative control strategy to enhance the search efficiency of ACO. Comparative analysis with several algorithms in the CEC 2017 benchmark functions confirms the superior performance of the IPCACO. Applying the bIPCACO-FKNN model for the prediction of pulmonary TSM achieved a prediction accuracy of 98.196% and a specificity of 99.500%, thus demonstrating its significant efficacy in accurately distinguishing between pulmonary TSM and tuberculosis. This provides an efficient and reliable machine learning tool for the differentiation between pulmonary TSM and tuberculosis.

## Highlights

• Introduced bIPCACO-FKNN model, integrating ant colony optimization with FKNN
• Enhanced search efficiency with incremental proportional-integral-derivative control
• Focused on non-HIV pulmonary TSM cases for model testing
• Achieved 98.196% accuracy in distinguishing pulmonary TSM from tuberculosis

## Graphical abstract

**Figure:**
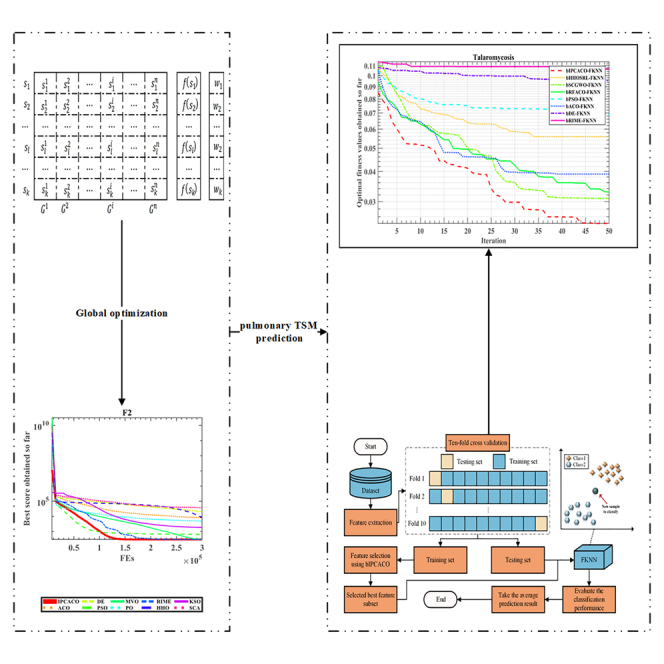

## Introduction

Talaromycosis (TSM) is a severe invasive disseminated fungal disease caused by *Talaromyces marneffei* (*T. marneffei*). Bamboo rats and humans are the most common animal hosts of *T. marneffei*, and humans can be infected through contact with bamboo rats. Soil exposure has also been reported to be associated with human infection, especially during the wet rainy season. The mode of infection may be direct inoculation or airborne transmission. The lungs are the most commonly affected organs in patients with TSM.^1^ A high incidence of TSM is predominantly observed in Southeast Asia and southern China, with a higher incidence among individuals infected with human immunodeficiency virus (HIV) or those who are severely immunosuppressed. However, there has been a consistent upward trend in the occurrence of TSM among HIV-negative patients since 2010.^1^ In recent years, TSM has emerged as the fastest-growing invasive pulmonary mycosis among HIV-negative individuals.^2^ Notably, TSM holds a prominent position in the World Health Organization’s inaugural list of fungal pathogens of major concern released in 2022.^3^

HIV-positive patients with TSM often present with an acute onset and severe clinical symptoms, and they have a high positive blood culture rate. Furthermore, previously, clinicians have focused on *T. marneffei* infection in HIV-positive patients, and this is relatively easy to diagnose. Conversely, HIV-negative patients with TSM exhibit normal CD4^+^ T lymphocyte counts and may not have any underlying disease. The clinical manifestations of this condition are complex and diverse and lack specificity. They can involve multiple organ systems throughout the body, including lung abnormalities, multiple systemic lymph node enlargement, osteolytic destruction and hepatosplenomegaly. Additionally, there is a significant lack of understanding among clinicians regarding the clinical manifestations, susceptibility factors, and imaging findings associated with HIV-negative TSM, making early diagnosis and treatment challenging. The initial diagnosis of pulmonary talaromycosis is frequently misdiagnosed as tuberculosis, pneumonia, lymphoma, or other malignant tumors characterized by extensive lymph node metastasis.^4^ Delayed diagnosis plays a crucial role in the high mortality rate and irreversible damage to organ function caused by TSM.^5^ The mortality rate of TSM in HIV-negative patients (27.7%–29.4%) was significantly greater than that in HIV-positive patients (20.7%–24.5%).^6^ Therefore, there is an unmet clinical need to improve the early identification and initiation of the diagnostic process for TSM, as well as prompt administration of antifungal treatment.

The detection of *T. marneffei* in sterile body fluids and tissue cultures serves as the gold standard for diagnosing TSM. This temperature-sensitive biphasic fungus requires specific culture conditions and a lengthy incubation period (typically 4–14 days, occasionally up to 26 days),^7^ making it challenging to establish an early diagnostic basis for clinical practice. Additionally, due to the low fungal load in HIV-negative individuals during the initial stages of infection, culture results may yield false-negatives, leading to missed diagnoses. Direct microscopic examination (including bone marrow smear, lung biopsy specimen, and lymph node biopsy tissue) and histopathology necessitate invasive procedures. Acquiring most specimens is challenging, and their interpretation requires experienced pathologists, making it difficult to meet the demands of early diagnosis. The use of a serum immunological test offers the advantage of rapid diagnostic capabilities. However, both the (1,3)-β-D-glucan (BDG) and galactomannan (GM) tests exhibit high sensitivity but low specificity. This is due to the potential for cross-reactivity with other pathogens, which limits the clinical applicability of these methods.^8^ Mp1p antigen detection has demonstrated non-cross-reactivity with common fungal pathogens, such as Cryptococcus, Candida, Aspergillus, and histoplasmosis, exhibiting high sensitivity and excellent specificity. Several studies have reported a sensitivity ranging from 75% to 86% and a specificity ranging from 98 to 99% for serum/plasma Mp1p antigen detection in diagnosing TSM. Paired plasma and urine detection can enhance diagnostic accuracy.^9^ However, the early diagnostic accuracy of Mp1p antigen testing in real clinical scenarios is unclear due to the lack of confirmatory studies supporting its clinical use. The PCR detection method is valuable for the early diagnosis of TSM and has a specificity of up to 100%. However, the sensitivity ranges from 70.4% to 91.0%.^10^ Owing to its substantial cost and demanding laboratory infrastructure requirements, this assay has not gained widespread use in clinical practice. The metagenomic next-generation sequencing (mNGS) method is a novel high-throughput pathogen detection technique based on molecular biology that enables rapid diagnosis by extracting all microbial nucleic acid sequences. It offers remarkable advantages in terms of heightened sensitivity and extensive coverage. However, challenges persist, including the absence of standardized diagnostic criteria and laboratory databases, uniform procedures, and exorbitant testing expenses.^11^ In summary, more rapid and effective diagnostic methods for assisting in clinical diagnosis and treatment still need to be explored and developed.

The lungs are the primary organs affected by *T. marneffei* infection. The majority of TSM exhibit abnormal chest imaging findings. Chest CT, the main diagnostic method for pulmonary diseases, offers advantages, such as rapidity, noninvasiveness, and repeatable. It provides a visual representation of lung involvement following *T. marneffei* infection and serves as an essential diagnostic tool for the early detection of pulmonary talaromycosis. However, the chest imaging manifestations of pulmonary talaromycosis are diverse and may include lung consolidation, cavities, miliary nodules, enlargement of hilar mediastinal lymph nodes, and pleural effusion. These manifestations can easily be mistaken for those seen in pulmonary tuberculosis and often pose challenges in distinguishing them with naked eye observation.^12^

It has been reported that the misdiagnosis rate of tuberculosis among all cases of TSM is as high as 80.77%.^13^ Recent advancements in artificial intelligence (AI) technology, coupled with robust data analysis and feature recognition capabilities, have the potential to offer doctors prompt and efficient assistance. The integration of AI with medical imaging technology has achieved significant breakthroughs in diagnosing certain diseases.^14^ Computer-aided diagnosis technology based on deep learning not only enhances the speed and accuracy of radiologists’ film interpretation but also facilitates self-learning and continuous optimization of interpretive outcomes.^15^ Currently, a series of studies have reported a number of AI diagnostic models for pulmonary diseases, such as lung nodules, lung cancer, and pneumonia, based on medical imaging techniques and the employment of diverse algorithms.^14^ Among these models, the diagnostic accuracy of AI models specifically developed for COVID-19 is exceptionally high, ranging from 94.0% to 95.2%.^16^ The image-based AI diagnostic model has played a pivotal role in distinguishing COVID-19 from community-acquired pneumonia and other respiratory conditions while facilitating triage at fever clinics, thereby significantly contributing to the prompt diagnosis of COVID-19.^17^

Huang et al.^18^ proposed a pulmonary embolism (PE) risk prediction model based on multiple machine learning (ML) methods, including logistic regression (LR), decision tree (DT), random forest (RandomF), naive bayes (NB), support vector machine (SVM), and adaptive boosting (AdaBoost). Experimental results demonstrated that the RF model exhibited higher predictive accuracy. Similarly, Xi et al.^19^ developed diagnostic models for acute PE using various ML techniques, such as RF, NB, Bayesian, DT, k-nearest neighbors (KNNs), LR, multi-layer perceptron (MLP), SVM, and gradient boosting decision tree (GBDT), with the RandomF-based model achieving the best performance. Yu et al.^20^ introduced an RF-based method for predicting in-hospital mortality among patients with acute exacerbations of chronic obstructive pulmonary diseases and compared its performance with existing models such as BAP-65 and CURB-65. Experimental results revealed that the RandomF model outperformed the BAP-65 and CURB-65 models in predictive capability. Bradley et al.^21^ utilized the Fibresolve ML software system for the non-invasive classification of idiopathic pulmonary fibrosis (IPF) and assessed its sensitivity in patients with non-definite usual interstitial pneumonia (UIP). Analysis of high-resolution computed tomography images showed that Fibresolve demonstrated high sensitivity in non-invasively identifying IPF among patients with non-definite UIP patterns. Ahamed et al.^22^ constructed a tuberculosis prediction model using multiple ML models, including DT, RandomF, SVM, and NB. Experimental results indicated that the DT model achieved superior predictive performance. Finally, Chen et al.^23^ proposed a deep neural network image classification algorithm based on Google Teachable Machine for predicting tuberculosis in chest X-rays. The experimental results demonstrated that the proposed model achieved high accuracy in detecting tuberculosis cases. Table 1 concludes the main work of these researches. Consequently, AI is increasingly assumed to play a crucial role in assisting with the diagnosis of various pulmonary diseases.

**Table 1:** Summary of related work about pulmonary diseases diagnosis

Reference	Main contribution
Huang et al.^18^	Employing different ML models (logistic regression, decision tree, random forest, naive Bayes, support vector machine, and adaptive boosting) to develop pulmonary embolism risk prediction model. Experimental results demonstrated that the RF model achieved the highest predictive accuracy.
Xi et al.^19^	Developing diagnostic models for acute PE based on various ML techniques. Among these proposed models, the random forest-based model exhibited the best performance.
Yu et al.^20^	Introducing a random forest-based method for predicting in-hospital mortality among patients with acute exacerbations of chronic obstructive pulmonary disease.
Bradley et al.^21^	Utilizing the Fibresolve ML system for non-invasive classification of idiopathic pulmonary fibrosis.
Ahamed et al.^22^	Constructing a tuberculosis prediction model using ML techniques. Experimental results indicated that the decision tree model achieved superior predictive performance.
Chen et al.^23^	Proposing a deep neural network-based image classification algorithm utilizing Google Teachable Machine for predicting tuberculosis in chest X-rays. Experimental results demonstrated that the proposed model achieved high accuracy in detecting tuberculosis cases.

Nonetheless, medical data often include a considerable quantity of extraneous information, which can impact the precision of predictive models. As a result, filtering out these irrelevant data is essential. Feature selection (FS) plays a vital role in data preprocessing, focusing on removing redundant features from the dataset to enhance model accuracy. FS can be divided into wrapper-based FS methods, filter-based FS methods, and embedded FS methods.^24^ Wrapper-based FS methods involve searching through feature subsets using a search strategy and using performance metrics of classifiers as evaluation criteria for these subsets. Thus, this method can identify more precise feature subsets, while at the cost of higher computational complexity. Filter-based FS methods, on the other hand, rank features based on their importance using feature importance evaluation techniques to select the most relevant ones. However, this method does not take into account the relationships between features. Embedded FS methods, on the other hand, directly consider the importance of features during the model training process for FS. Nevertheless, this method is constrained by the limitations of the selected classification model.

When considering all feature subsets through exhaustive enumeration, the solution space grows exponentially with the increasing number of features.^25^ Therefore, FS is regarded as an NP-hard problem. As it is impractical to search through all combinations of the feature space, more and more researchers are turning to various metaheuristic algorithms (Mas) for wrapper-based FS methods. Mas are a class of optimization algorithms that gradually approach the global optimal solution in the solution space by simulating natural phenomena or behaviors. Common Mas algorithms include differential evolution (DE),^26^ ant colony optimization (ACO),^27^ particle swarm optimization (PSO),^28^ Runge Kutta optimization (RUN),^29^ rime optimization algorithm (RIME),^30^ Harris hawks optimization (HHO),^31^ and liver cancer algorithm (LCA),^32^ among others. ACO, inspired by the foraging behavior of ants, is one such Mas algorithm. Due to its flexibility and effectiveness, it has been widely applied in various optimization problems.^33^^,^^34^^,^^35^

In this study, aimed at assisting physicians in diagnosing TSM and pulmonary tuberculosis, a classification model for TSM is proposed based on CT scan image features collected from 144 cases, including HIV-negative pulmonary TSM patients and HIV-negative pulmonary tuberculosis patients. The model integrates ACO and fuzzy k-nearest neighbor (FKNN) techniques. Although the original ACO exhibits good optimization capabilities, it still has its limitations. For instance, in the original ACO framework, individual position updates rely solely on a subset of optimal solutions, neglecting the positions of other individuals within the population. This oversight may lead the algorithm to converge prematurely to local optima. As a result, this study initially introduces a modified version of ACO, based on the incremental proportional-integral-derivative (PID) control search (IPC) strategy, named IPCACO, to improve the optimization capabilities of the original ACO. To validate the effectiveness of IPCACO, experimental comparisons are conducted with the original ACO and state-of-the-art (SOTA) algorithms using the CEC 2017 benchmark test functions. Subsequently, based on IPCACO, a wrapper-based FS method using FKNN as the classifier (bIPACO-FKNN) is proposed to filter out key features from 144 collected cases, aiming to improve the accuracy of the TSM classification model. Further validation of the proposed FS method is conducted comparing with similar methods. Finally, experimental results of the proposed method in the actual clinical diagnosis of TSM are discussed. The main contributions of this paper are as follows.

(1) An enhanced variant of ACO is proposed. The IPC strategy dynamically adjusts the updating policy of individual positions during the updating process based on current positional information. This adaptation preserves population diversity, thereby enhancing the algorithm’s convergence capability.
(2) A wrapper-based FS TSM prediction model is proposed utilizing IPCACO and FKNN.
(3) Comparative experiments with similar methods are carried out on the CEC 2017 benchmark data and the collected TSM dataset to verify the performance of the proposed method. The findings illustrate the method’s effectiveness in discovering optimal solutions.

## Results

### Experiments on benchmark functions

This section presents the optimization results of IPCACO on the CEC 2017 benchmark functions and compares the results with those of nine original algorithms and eleven SOTA algorithms to comprehensively analyze the optimization performance of IPCACO.

### Experiment setup

The CEC 2017 benchmark functions^36^ comprises a total of 29 test functions, among which F1 and F2 are unimodal functions, F3–F9 are multimodal functions, F10–F19 are hybrid functions, and F20–F29 are composition functions. These functions simulate a search space containing numerous local optima, thus providing a robust evaluation of algorithms’ capabilities to find the global optimum. Details of the CEC 2017 benchmark functions are provided in Table 2, with the upper and lower bounds of the benchmark test functions set at ±100, and the dimensionality fixed at 30.

**Table 2:** The details of CEC 2017 benchmark functions

ID	Name of the function	Class	Optimum
$f_{1}$	Shifted and Rotated Bent Cigar Function	Unimodal	100
$f_{2}$	Shifted and Rotated Zakharov Function		300
$f_{3}$	Shifted and Rotated Rosenbrock’s Function	Multimodal	400
$f_{4}$	Shifted and Rotated Rastrigin’s Function		500
$f_{5}$	Shifted and Rotated Expanded Scaffer’s F6 function		600
$f_{6}$	Shifted and Rotated Lunacek Bi-Rastrigin Function		700
$f_{7}$	Shifted and Rotated Non-Continuous Rastrigin’s Function		800
$f_{8}$	Shifted and Rotated Lévy Function		900
$f_{9}$	Shifted and Rotated Schwefel’s Function		1000
$f_{10}$	Hybrid Function 1 (*N* = 3)	Hybrid	1100
$f_{11}$	Hybrid Function 2 (*N* = 3)		1200
$f_{12}$	Hybrid Function 3 (*N* = 3)		1300
$f_{13}$	Hybrid Function 4 (*N* = 4)		1400
$f_{14}$	Hybrid Function 5 (*N* = 4)		1500
$f_{15}$	Hybrid Function 6 (*N* = 4)		1600
$f_{16}$	Hybrid Function 6 (*N* = 5)		1700
$f_{17}$	Hybrid Function 6 (*N* = 5)		1800
$f_{18}$	Hybrid Function 6 (*N* = 5)		1900
$f_{19}$	Hybrid Function 6 (*N* = 6)		2000
$f_{20}$	Composition Function 1 (*N* = 3)	Composition	2100
$f_{21}$	Composition Function 2 (*N* = 3)		2200
$f_{22}$	Composition Function 3 (*N* = 4)		2300
$f_{23}$	Composition Function 4 (*N* = 4)		2400
$f_{24}$	Composition Function 5 (*N* = 5)		2500
$f_{25}$	Composition Function 6 (*N* = 5)		2600
$f_{26}$	Composition Function 7 (*N* = 6)		2700
$f_{27}$	Composition Function 8 (*N* = 6)		2800
$f_{28}$	Composition Function 9 (*N* = 3)		2900
$f_{29}$	Composition Function 10 (*N* = 3)		3000

To validate the performance of IPCACO, nine original algorithms including ACO,^27^ DE,^26^ PSO,^28^ multi-verse optimization (MVO),^37^ parrot optimizer (PO),^38^ RIME,^30^ HHO,^31^ kernel search optimizer (KSO),^39^ and sine cosine algorithm (SCA),^40^ as well as eleven SOTA algorithms, namely fruit fly optimization algorithm enhanced by the Levy flight (MALBFOA),^41^ adaptive SCA integrated with PSO (ASCA-PSO),^42^ hierarchical guided SMA (HG_SMA),^43^ fuzzy self-tuning PSO (FSTPSO),^44^ chaotic arc adaptive grasshopper optimization (CGSCA),^45^ CGPSO,^46^ an enhanced whale optimization algorithm (SCDWOA),^47^ mutational SMA (ISMA),^48^ a double adaptive weight mechanism enhanced month-flame optimization (WEMFO),^49^ gravitational search algorithm with self-adaptive gravitational constants (ALGSA),^50^ and a boosted PSO (SRWPSO),^51^ are selected for comparison with IPCACO. The population size for all algorithms is fixed at 30, with a maximum of 300,000 evaluations. Additional parameter settings are provided in Tables 3 and 4. To ensure a fair comparison, each algorithm was run independently 30 times. The performance was then assessed by comparing the average (Avg) and standard deviation (SD) of the fitness values from these 30 runs.

**Table 3:** The parameter setting of original Mas

Algorithm	Parameter information
IPCACO	q=0.7;ibslo=0.8;beta=1.5
ACO	k=9;q=0.6;ibslo=1
PSO	wMax=0.9;wMin=0.2;c1=2;c2=2.
DE	pCR=0.2;beta_min=0.2;beta_max=0.8.
MVO	WEP_Max=1;WEP_Min=0.2
PO	\
RIME	W=5
HHO	r=[0,1]
KSO	\
SCA	a=2

**Table 4:** The parameter setting of SOTA algorithms

Algorithm	Parameter information
IPCACO	q=0.7;ibslo=0.8;beta=1.5
CGPSO	1=c2=2;W_max=0.9;W_min=0;W_min=0.2;V_max=6
ASCA-PSO	M=4;N=9;Vmax=6;wMax=0.9;wMin=0.2;c1=2;c2=2
ALGSA	p=0.5;limit=2
HG_SMA	Elite_num=10;c1=0.5;c2=2;w=1.4
FSTPSO	Vmax=6;Vmin=−6;wMax=0.9;wMin=0.2;c1=2;c2=2;w=0.9
CGSCA	a=2;delta=0.1
WEMFO	b=1
MALBFOA	pa=6;M=10
ISMA	beta_min=0.2;beta_max=0.8;pCR=0.8;z=0.03
SCDWOA	gapm=5;r=[0,1];b=1
SRWPSO	wMax=0.9;wMin=0.2;c1=2;c2=2;Vmax=6

Additionally, to validate the statistical significance of IPCACO, the Wilcoxon signed-rank test (WST),^52^ Friedman test (FT)^53^ with Bonferroni-Dunn post-hoc test (BPT)^54^ will be employed. These statistical tests aim to demonstrate the superiority of IPCACO in terms of statistical significance.

### Comparison with original Mas

**Figure 1:**
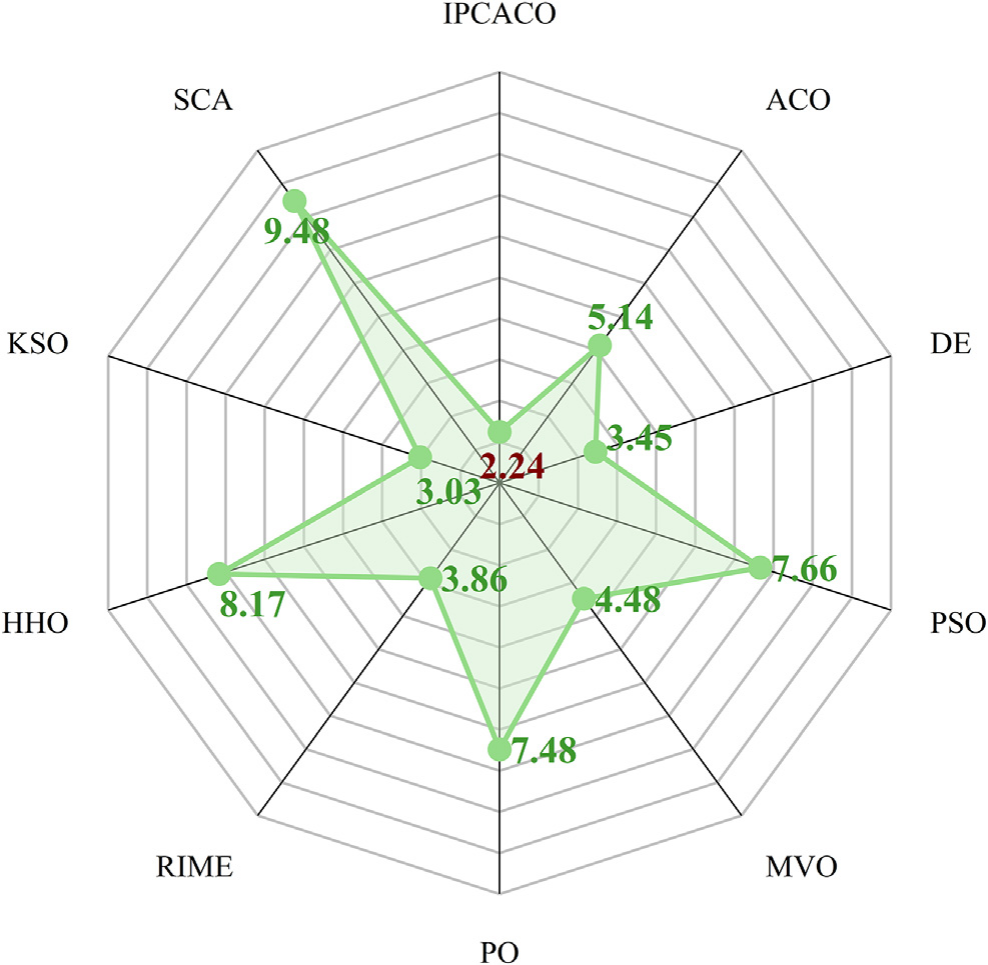
Average rank result of IPCACO and comparative algorithms based on Avg and SD

In this section, IPCACO is evaluated against the algorithms listed in Table 3 using the CEC 2017 benchmark functions. Table S1 shows the Avg and SD achieved by all algorithms on the test functions. While IPCACO attained the top ranking in seven test functions (F2, F3, F10, F13, F14, F23, F27, and F29), it ranked second in thirteen others. Figure 1 displays the average ranking results based on Avg and SD. IPCACO achieved the highest overall ranking with an average rank of 2.24, followed by KSO and DE. This demonstrates IPCACO’s strong optimization capability.

**Figure 2:**
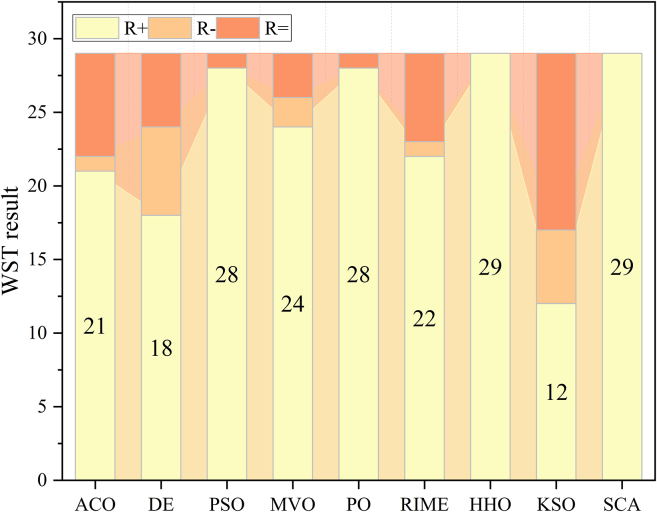
WST result of IPCACO and comparative algorithms

Figure 2 illustrates the performance of IPCACO compared to the other algorithms on the WST. In the figure, ‘R+', ‘R-', and ‘R = ' represent the number of test functions where IPCACO’s optimization performance is better than, worse than, or comparable to that of the comparison algorithms. The results demonstrate that IPCACO generally surpasses the other algorithms in most of the test functions. In this test, the algorithm closest in performance to the proposed IPCACO is KSO, with IPCACO outperforming KSO in 12 out of 29 functions. Table S2 records the *p* values obtained by IPCACO and the comparison algorithms on the WST. The *p* values for most test functions in the table are less than 0.05, indicating significant differences between IPCACO and the comparison algorithms.

**Figure 3:**
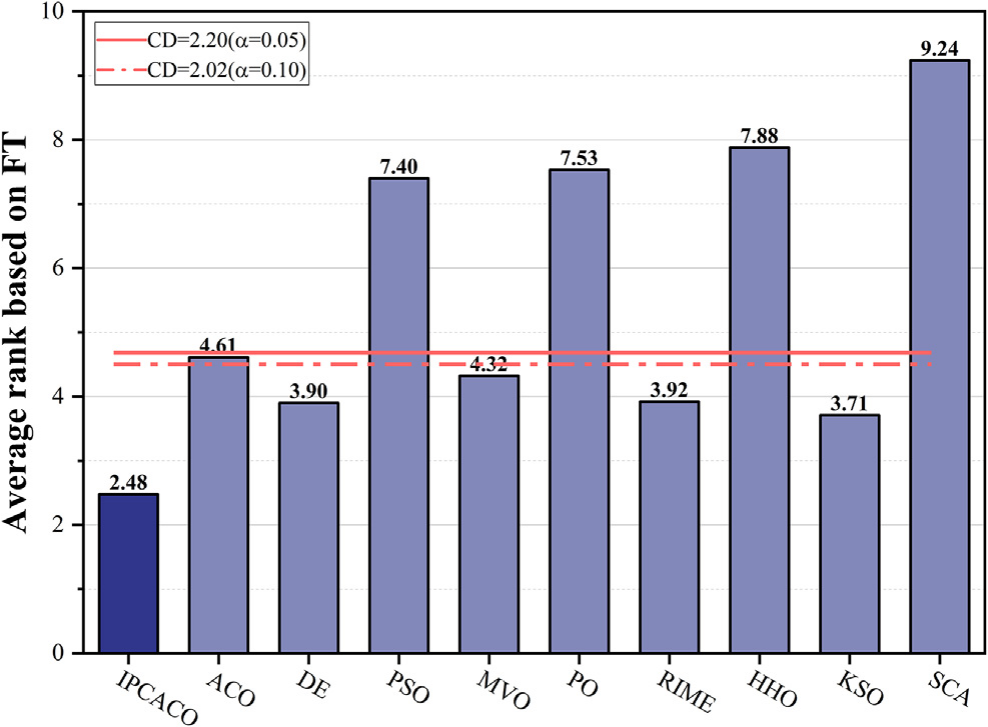
BPT result of IPCACO and comparative algorithms

Table 5 displays the average ranking results of IPCACO and the comparison algorithms on FRT. A smaller average ranking value indicates better algorithm performance. The results in the table indicate that IPCACO achieves the top ranking in average results, followed by KSO and DE, demonstrating the effectiveness of IPCACO. Based on the FRT results, BPT is conducted to explore whether significant differences exist between IPCACO and the comparison algorithms, as shown in Figure 3. When the FRT average ranking of a comparison algorithm surpasses the threshold line, it indicates a significant difference in performance between IPCACO and the comparison algorithm at the current significance level. According to the figure, IPCACO shows notable performance differences from PSOMPO, HHO, and SCA at both significance levels. However, there are no significant performance differences between IPCACO and DE, MVO, RIME, and KSO.

**Table 5:** Friedman average rank of IPCACO and comparative algorithms

Algorithm	AVR	Rank	Algorithm	AVR	Rank
IPCACO	2.48	1	PO	7.53	8
ACO	4.61	6	RIME	3.92	4
DE	3.90	3	HHO	7.88	9
PSO	7.40	7	KSO	3.71	2
MVO	4.32	5	SCA	9.24	10

**Figure 4:**
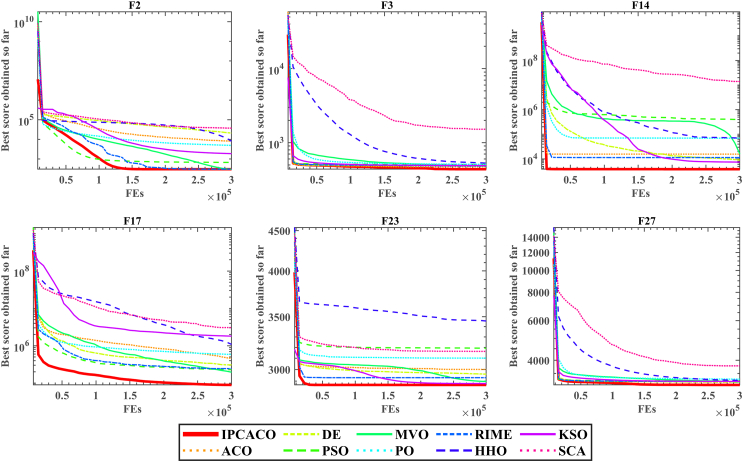
Convergence curves of IPCACO and comparative algorithms

Figure 4 presents the convergence curves of IPCACO and comparison algorithms on selected functions. It can be observed that IPCACO exhibits faster convergence speed. In functions F3, F14, F23, and F27, IPCACO not only converges rapidly in the early evaluations but also achieves the highest convergence accuracy. In functions F2 and F17, IPCACO demonstrates superior convergence speed in the early evaluations compared to the comparison algorithms. Furthermore, in the later evaluations, IPCACO avoids being trapped in local optima and continues to converge to better positions. The reason why IPCACO has better convergence ability than the comparison algorithm is due to the introduction of PID control idea, so the algorithm can adaptively adjust the search direction during the solution search process. This adaptive adjustment makes the algorithm have better ability to jump out of the local optimum, so it has better convergence ability.

**Figure 5:**
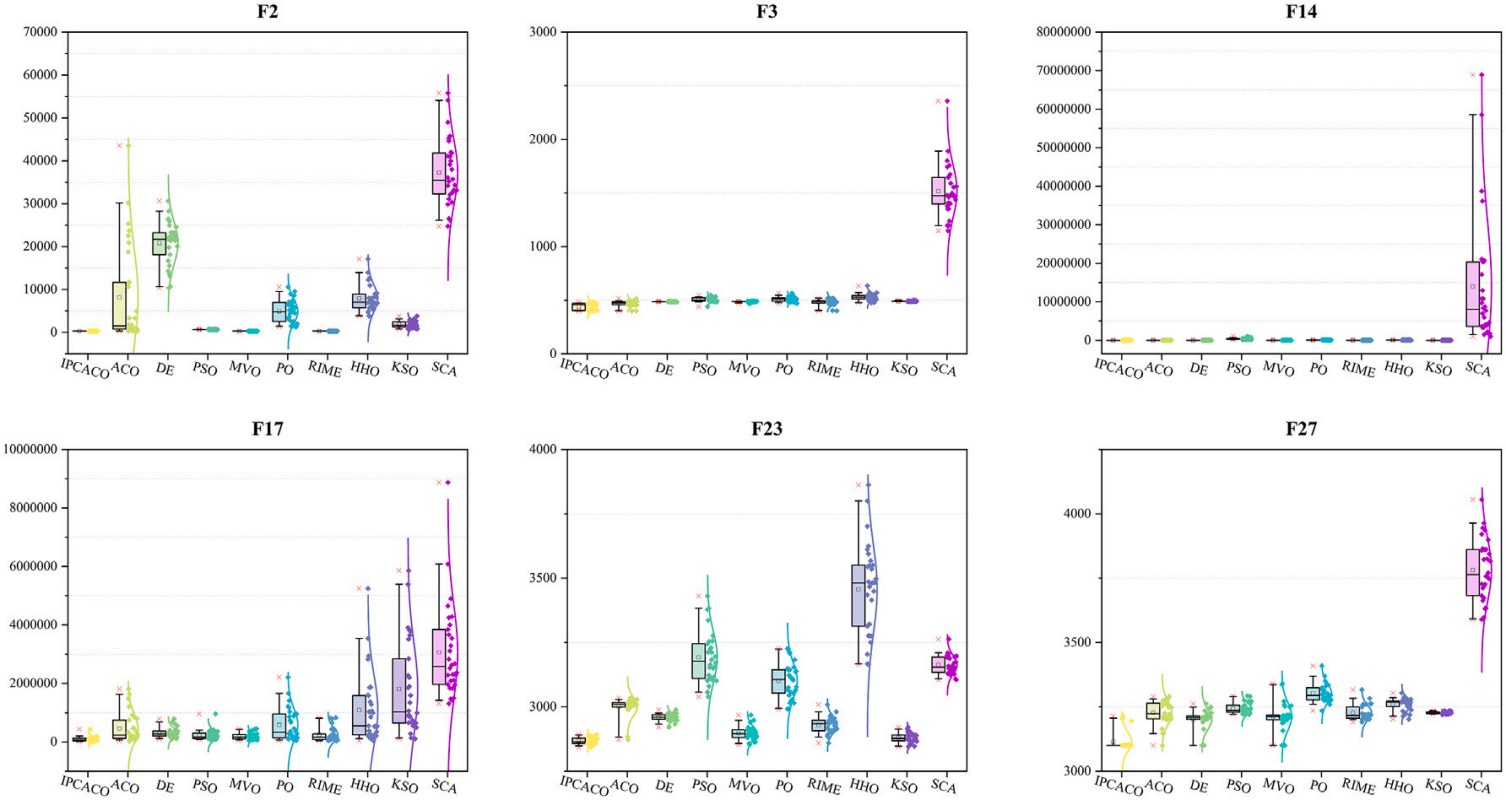
Boxplots of IPCACO and comparative algorithms

Figure 5 displays boxplots illustrating the optimization results of each algorithm on selected test functions. The data presented show that IPCACO achieves higher-quality solutions with less variability compared to the other algorithms. Overall, IPCACO demonstrates better convergence capabilities in comparison to the other algorithms.

### Comparison with SOTA algorithms

**Figure 6:**
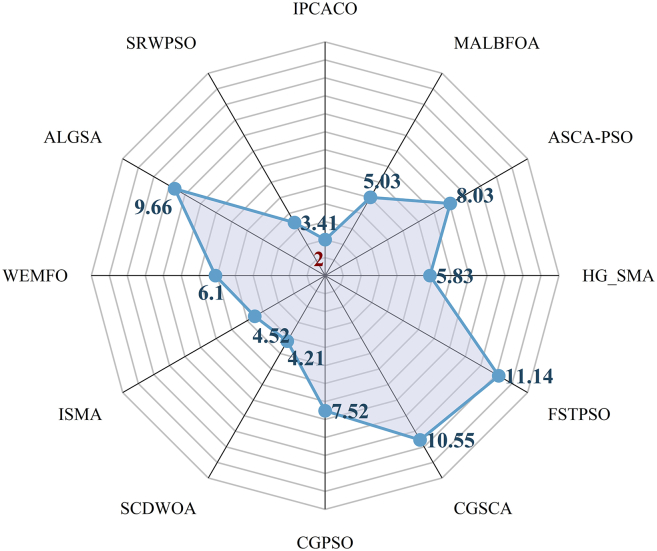
Average rank result of IPCACO and SOTA algorithms based on Avg and SD

In this section, IPCACO is evaluated against 11 state-of-the-art algorithms detailed in Table 4 using the CEC 2017 benchmark functions. Table S3 provides the Avg and SD for each algorithm on the test functions. The results show that IPCACO outperforms the comparison algorithms, proving to be the most effective method for solving functions F4-F8, F10, F15, F16, F19, F20, F22, F23, F25, and F28. IPCACO also achieved favorable ranking results on the remaining test functions. Figure 6 provides the average ranking values of each algorithm, where it can be observed that the proposed IPCACO ranks first with an Avg of 2, followed by SRWPSO and SCDWOA.

**Figure 7:**
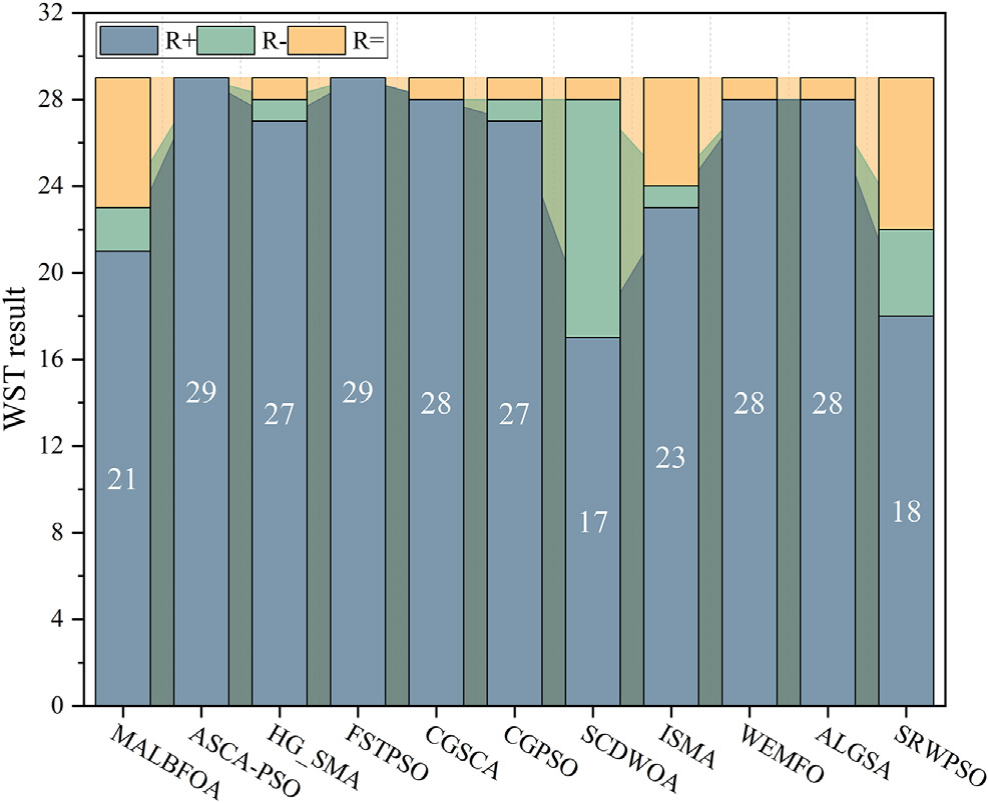
WST result of IPCACO and SOTA algorithms

**Figure 8:**
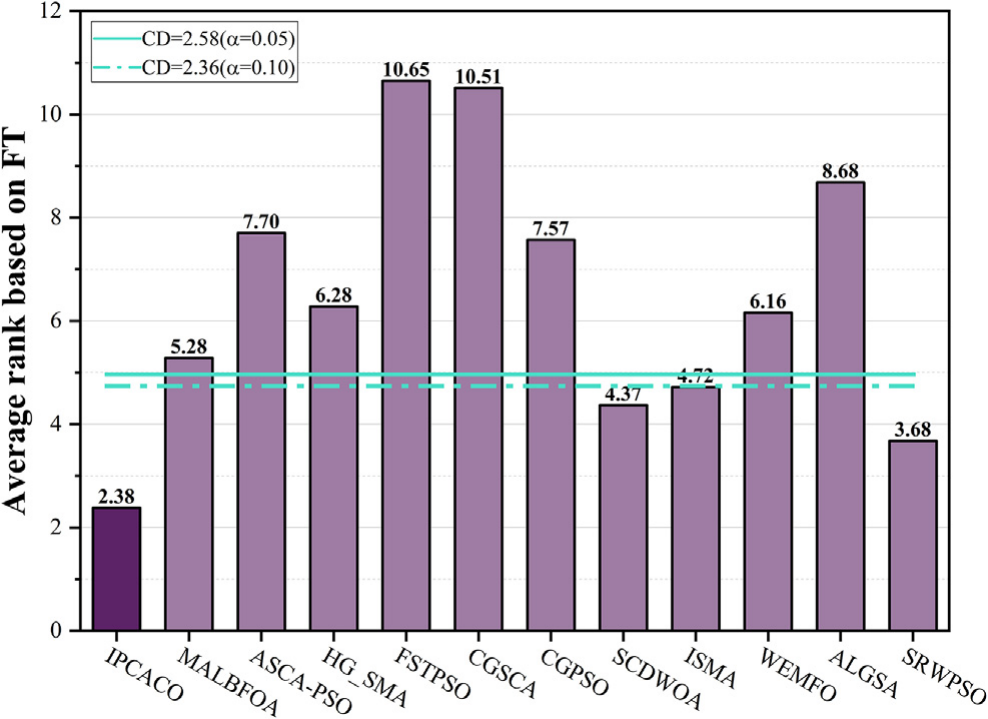
BPT result of IPCACO and SOTA algorithms

The results obtained from the WST are shown in Figure 7. From the figure, it can be observed that IPCACO’s performance is significantly better than ASCA-PSO and FSTPSO on all test functions. Additionally, IPCACO outperforms CGSCA, WEMFO, and ALGSA on 28 test functions. Table S4 contains the *p* values derived from the WST. Most of these *p* values are below 0.05, which supports the superior performance of IPCACO. Additionally, Table 6 shows the average ranking values calculated from the FRT results. The table reveals that IPCACO achieves the highest rank, demonstrating its strong competitive edge. Subsequently, based on these FRT values, BPT is conducted for IPCACO and the SOTA algorithms, and the results are depicted in Figure 8. Based on the results depicted in the figure, it can be concluded that IPCACO significantly differs from MALBFOA, ASCA-PSO, HG_SMA, FSTPSO, CGSCA, WEMFO, and ALGSA at both significance levels. Conversely, there are no significant differences between IPCACO and SCDWOA, ISMA, and SRWPSO.

**Table 6:** Friedman average rank of IPCACO and SOTA algorithms

Algorithm	AVR	Rank	Algorithm	AVR	Rank
IPCACO	2.38	1	CGPSO	7.57	8
MALBFOA	5.28	5	SCDWOA	4.37	3
ASCA-PSO	7.70	9	ISMA	4.72	4
HG_SMA	6.28	7	WEMFO	6.16	6
FSTPSO	10.65	12	ALGSA	8.68	10
CGSCA	10.51	11	SRWPSO	3.68	2

**Figure 9:**
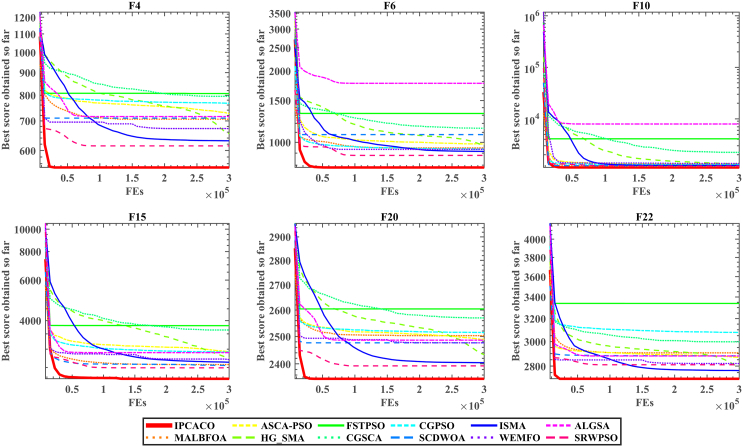
Convergence curves of IPCACO and SOTA algorithms

**Figure 10:**
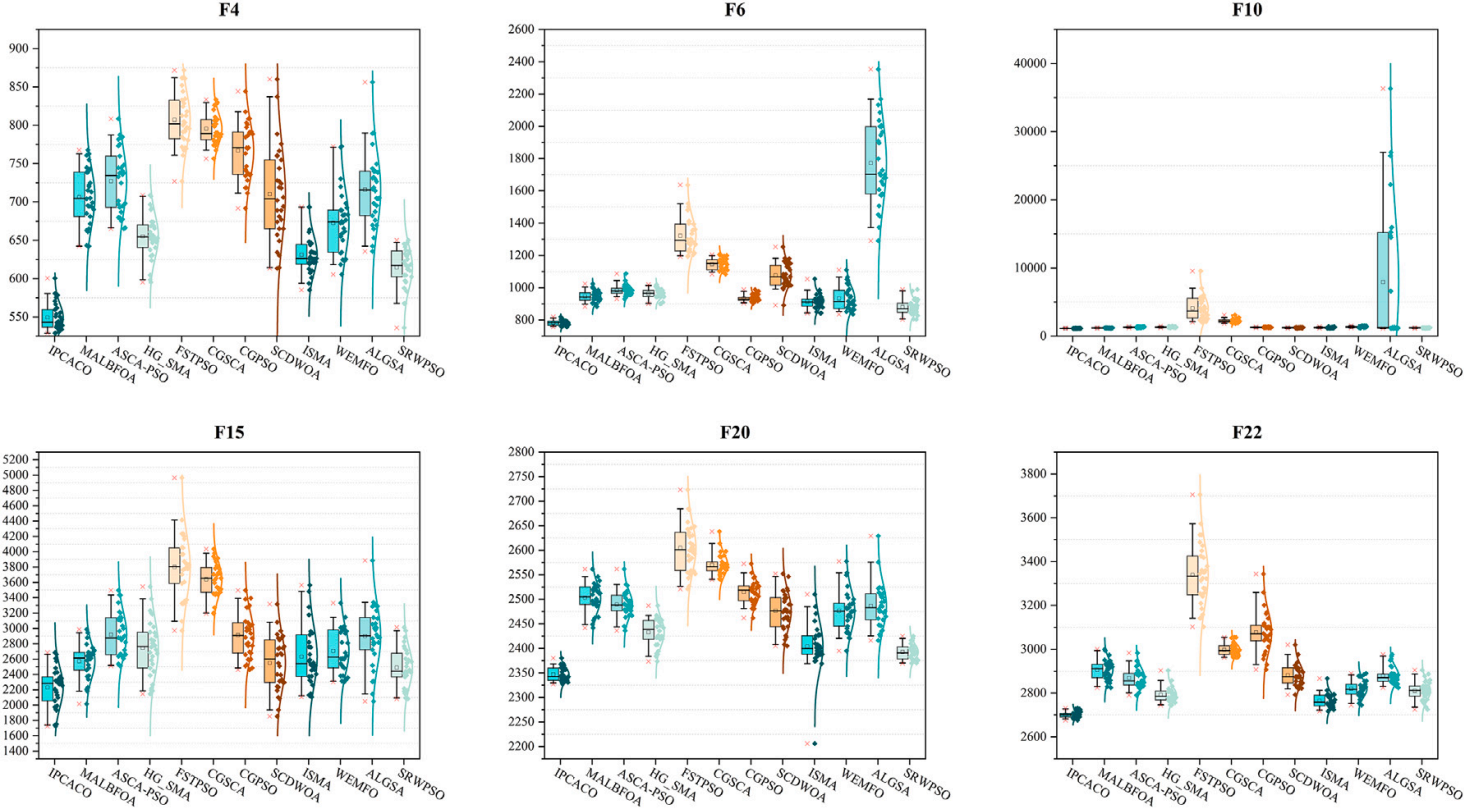
Boxplots of IPCACO and SOTA algorithms

Figures 9 and 10 present the convergence curves and boxplots for IPCACO and state-of-the-art algorithms on various test functions. The data clearly show that IPCACO achieves both the quickest convergence speed and the highest accuracy on these functions. This suggests that the IPC strategy significantly boosts the convergence speed of the original ACO while also delivering superior convergence accuracy. Moreover, IPCACO’s distribution in the boxplot of Figure 10 is more concentrated, with lower values, indicating its better stability.

### Talaromycosis prediction experiments

#### Evaluation criteria

In conducting predictive experiment on talaromycosis, a standardized experimental framework was implemented to ensure the consistency of experimental conditions. The predictive efficacy was assessed through a series of experimental operations. During the experiments, the FS algorithm used a population size of 20, with the data dimensionality adjusted to meet the specific needs of the talaromycosis dataset. Additionally, the algorithm was set to run for 50 iterations. To reduce the effects of random experimental errors, ten independent rounds of testing were performed. The proposed model’s performance was assessed using six evaluation metrics: accuracy, sensitivity, specificity, precision, Matthews correlation coefficient (MCC), and F-score, to gauge its prediction accuracy and effectiveness. The Avg and SD of these metrics were calculated. The mathematical formulations for these evaluative criteria are detailed in Table 7.

**Table 7:** Specific information on evaluation metrics

Metrics	Calculation formula
Accuracy	$Accuracy=\frac{TN+TP}{TN+FP+TP+FN}$
Sensitivity	$Sensitivity=\frac{TP}{TP+FN}$
Specificity	$Specificity=\frac{TN}{TN+FP}$
Precision	$Precision=\frac{TP}{TP+FP}$
MCC	$MCC=\frac{TP\times TN-FP\times FN}{\sqrt{\left(TP+FP\right)\times \left(TP+FN\right)\times \left(TN+FP\right)\times \left(TN+FN\right)}}$
F-measure	$F-measure=\frac{TP}{TP+\left(FN+FP\right)/2}$

TP, true positive; FN, false negative; FP, false positive; TN, true negative.

#### Results and analysis of talaromycosis prediction

This section outlines an experimental investigation conducted in three phases. Initially, a variety of classifiers are amalgamated with the proposed bIPCACO FS algorithm to ascertain the classifier most apt for the prediction of TSM. Subsequent to the determination of the optimal classifier, an evaluation of different TFs is conducted, with the aim of selecting the premier TF for the accurate prediction of TSM. Ultimately, upon the identification of the optimal classifier and TF, this superior model is juxtaposed with existing models, with the intention of substantiating the enhancements and advantages of the new model in the prediction of TSM.

#### Classifier design for feature selection models

Within a multitude of domains, including but not limited to disease prediction,^55^ financial analysis,^56^ and intrusion detection,^57^ algorithms based on wrapper FS have found extensive application. The principal aim of such algorithms is to filter the original feature set to construct an optimized subset of features, with the intention of significantly enhancing the overall performance of the model. However, the efficiency of wrapper FS algorithms is largely contingent upon the accurate assessment of the contribution of the selected feature subset to the classifier’s performance. Therefore, in the process of employing a wrapper FS algorithm for the prediction of TSM, selecting an efficient and suitable classifier becomes particularly critical.

To explore this issue, the FS algorithm proposed in this study (bIPCACO) was integrated with various classifiers, specifically including kernel extreme learning machine (KELM), FKNN, MLP, SVM, and KNN, subsequently forming bIPCACO-KELM, bIPCACO-FKNN, bIPCACO-MLP, bIPCACO-SVM, and bIPCACO-KNN. Through the evaluation of these models’ predictive performance on TSM, the objective was to identify the classifier most suitable for the TSM prediction task. Detailed configurations of the model parameters for these five models, which incorporate the wrapper FS method, are provided in Table 8.

**Table 8:** Parameter settings for the five methods

Methods	Parameter values
bIPCACO-KELM	k=1;m=2
bIPCACO-FKNN	c=88;γ=1024
bIPCACO-MLP	hiddenlayer=50;learningrate=0.01
bIPCACO-SVM	c=850;γ=0.17
bIPCACO-KNN	k=1

**Figure 11:**
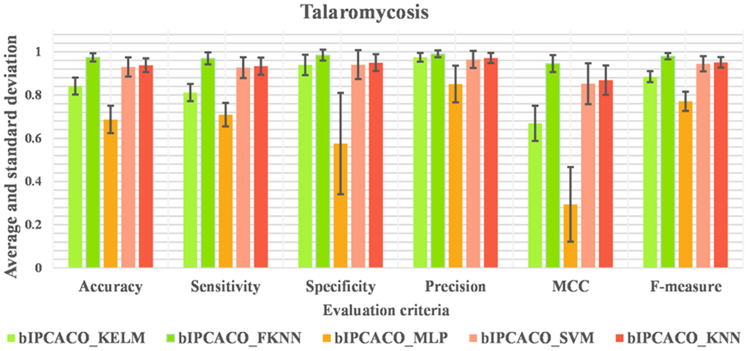
Comparison of bIPCACO combining different classifiers for TSM prediction

Table 9 presents the Avg and SD performance metrics of the bIPCACO algorithm integrated with different classifiers for TSM prediction. Figure 11 offers a more intuitive comparison of the bIPCACO algorithm’s performance across various classifiers in predicting TSM. From the perspective of accuracy, the bIPCACO-FKNN model exhibits superior performance, achieving an average accuracy of 97.392% with a minimal SD of 0.019%, indicating exceptionally high stability and consistency across different runs. In contrast, the bIPCACO-MLP model performs less adequately, with an average accuracy of only 68.653% and a standard deviation of 0.064%, reflecting its weaker predictive capability and stability relative to other models.

**Table 9:** Avg and SD of bIPCACO combining different classifiers for TSM prediction

Methods	Accuracy		Sensitivity		Specificity	
	Avg	SD	Avg	SD	Avg	SD
bIPCACO-KELM	84.151%	0.039	81.120%	0.040	93.938%	0.047
bIPCACO-FKNN	97.392%	0.019	96.929%	0.028	98.418%	0.026
bIPCACO-MLP	68.653%	0.064	70.943%	0.054	57.550%	0.234
bIPCACO-SVM	92.963%	0.044	92.686%	0.048	94.022%	0.066
bIPCACO-KNN	93.784%	0.032	93.339%	0.040	94.986%	0.039

Methods	Precision		MCC		F-measure	
	Avg	SD	Avg	SD	Avg	SD
bIPCACO-KELM	97.419%	0.020	0.66892	0.081	88.465%	0.025
bIPCACO-FKNN	99.032%	0.016	0.94532	0.040	97.941%	0.015
bIPCACO-MLP	85.108%	0.085	0.29400	0.172	77.088%	0.045
bIPCACO-SVM	96.419%	0.039	0.85214	0.095	94.451%	0.035
bIPCACO-KNN	97.097%	0.024	0.86849	0.068	95.131%	0.024

In terms of sensitivity, the bIPCACO-FKNN model also excels, with an average of 96.929%, slightly below its accuracy rate but still leading among all models. For the specificity metric (the ability to identify negative classes), the bIPCACO-FKNN model once again takes the lead, with an average specificity of 98.418% and a standard deviation of 0.026%, showcasing its exceptional capability in distinguishing non-TSM samples. The bIPCACO-MLP model shows the lowest specificity at 57.550%, with a higher standard deviation (0.234%), indicating unstable performance and a propensity for false positives in identifying negative class samples.

Precision, MCC, and F-measure metrics further corroborate the findings aforementioned. The bIPCACO-FKNN model demonstrates superior performance across all these metrics, especially notable in MCC (0.94532) and F-measure (97.941%), significantly surpassing other models. This reflects its excellence in predictive accuracy and the ability to balance the prediction capabilities for both positive and negative classes. Conversely, the bIPCACO-MLP model significantly lags behind other models in these three metrics, particularly in MCC, scoring only 29.400%, indicating the inferior quality of its prediction outcomes.

**Figure 12:**
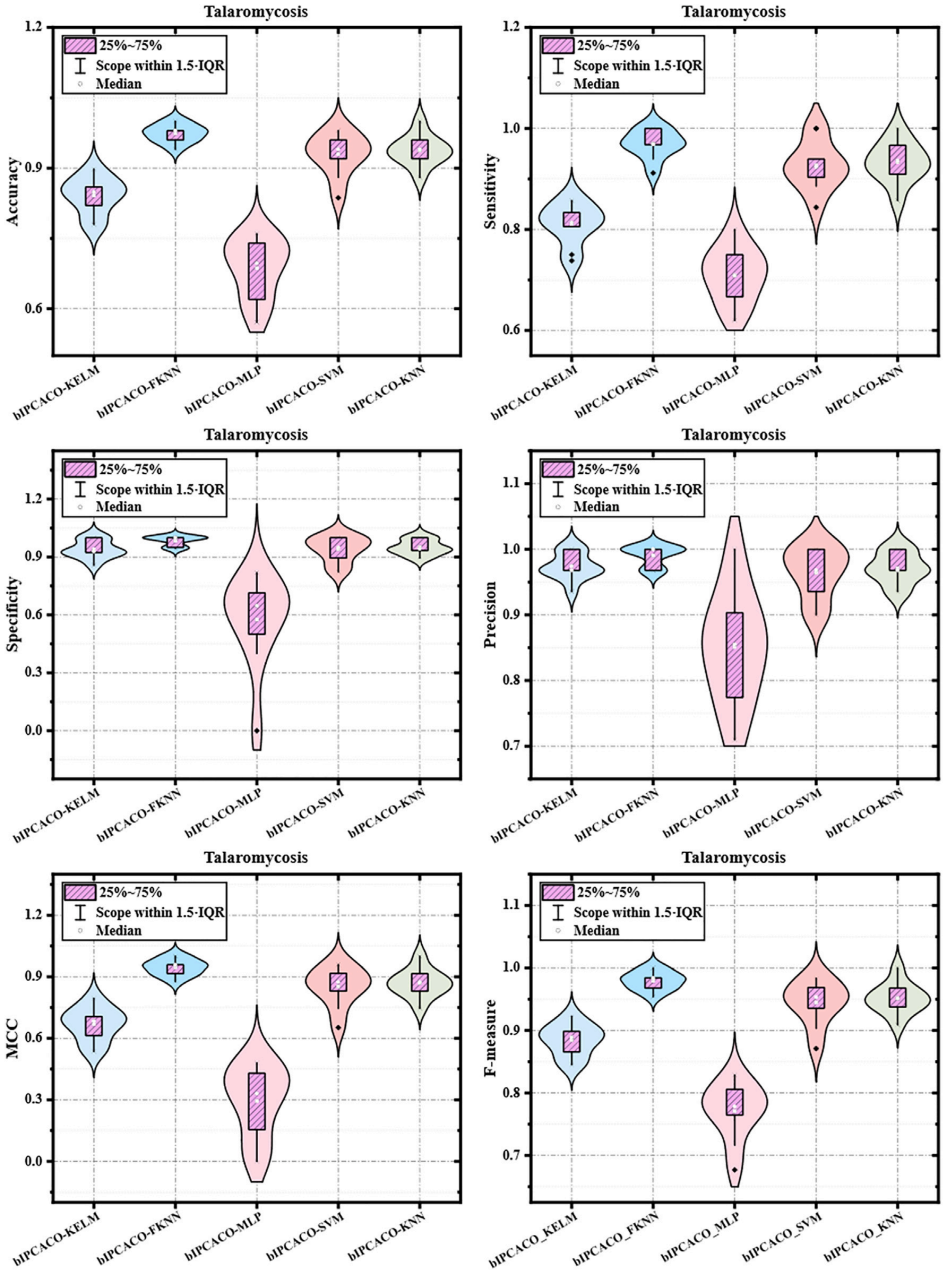
Distribution of evaluation metric values of bIPCACO combining different classifiers on the prediction of TSM

Additionally, Figure 12 displays the distribution of metric values across 10 independent experiments for TSM prediction using the bIPCACO algorithm with different classifiers. It is evident that, comparatively, the stability of the bIPCACO-FKNN, bIPCACO-KELM, bIPCACO-SVM, and bIPCACO-KNN models is relatively higher, whereas the predictive stability of the bIPCACO-MLP model requires improvement.

In summary, the bIPCACO-FKNN model outperforms in the TSM prediction task, exhibiting high accuracy, stability, and excellent classification performance. In contrast, the bIPCACO-MLP model shows inferior performance across multiple metrics, necessitating further improvement and optimization. Other models like bIPCACO-KELM, bIPCACO-SVM, and bIPCACO-KNN also demonstrate commendable performance but still have room for improvement on certain metrics.

#### Transfer function design for feature selection models

Recent studies have unveiled that the employment of various TFs in disease prediction exercises a discernible influence on the accuracy and consistency of the predictive outcomes.^47^^,^^58^ Following the determination that the FKNN classifier is the most apt for the bIPCACO FS algorithm in predicting TSM, hence constituting the bIPCACO-FKNN model, research further delved into the efficacy of integrating different TFs with this model. This exploration included combining the bIPCACO-FKNN model with a multitude of TFs, encompassing S-shaped TFs and V-shaped TFs (for detailed delineation, refer to Table 10). The objective was to identify the optimal TF that could maximally enhance the predictive performance of the bIPCACO-FKNN model in forecasting TSM.

**Table 10:** Details of the TF

Transfer function			
S-shaped TF		V-shaped TF	
Style	Specific formula	Style	Specific formula
S1	$T\left(x\right)=\frac{1}{1+e^{-2x}}$	V1	$T\left(x\right)=\left|\frac{\sqrt{2}}{\pi }\int_{0}^{\left(\sqrt{\pi }/2\right)x}e^{-t^{2}}dt\right|$
S2	$T\left(x\right)=\frac{1}{1+e^{-x}}$	V2	T(x)=|tanhx|
S3	$T\left(x\right)=\frac{1}{1+e^{-x/2}}$	V3	$T\left(x\right)=\left|x/\sqrt{1+x^{2}}\right|$
S4	$T\left(x\right)=\frac{1}{1+e^{-x/3}}$	V4	$T\left(x\right)=\left|\frac{2}{\pi }\arctan\left(\frac{\pi }{2}x\right)\right|$

**Figure 13:**
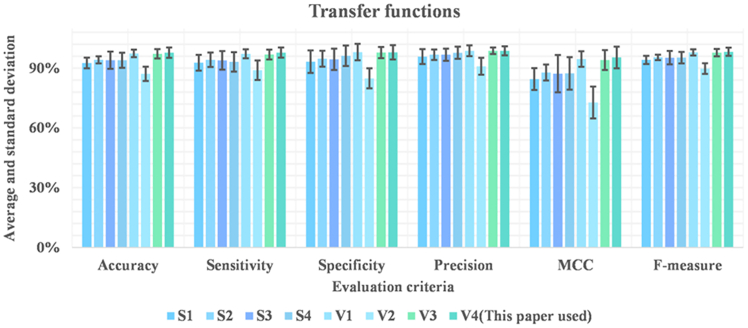
Performance comparison of bIPCACO-FKNN in predicting TSM on different TFs

Table 11 displays the Avg and SD performance metrics of the bIPCACO-FKNN model for predicting TSM when integrated with different TFs. These metrics encompass accuracy, sensitivity, specificity, precision, MCC, and F-measure. Figure 13 offers a more intuitive comparison of the bIPCACO-FKNN model’s performance in predicting TSM across various TFs.

**Table 11:** Avg and SD of the bIPCACO-FKNN for predicting TSM at different TFs

Methods	Accuracy		Sensitivity		Specificity	
	Avg	SD	Avg	SD	Avg	SD
bIPCACO-FKNN-S1	92.576%	0.027	92.732%	0.039	93.276%	0.056
bIPCACO-FKNN-S2	94.167%	0.018	94.192%	0.036	94.738%	0.040
bIPCACO-FKNN-S3	93.980%	0.043	93.897%	0.047	94.440%	0.054
bIPCACO-FKNN-S4	93.958%	0.038	93.137%	0.049	96.219%	0.052
bIPCACO-FKNN-V1	97.392%	0.019	97.194%	0.023	98.048%	0.041
bIPCACO-FKNN-V2	87.139%	0.036	88.967%	0.049	84.853%	0.050
bIPCACO-FKNN-V3	97.192%	0.023	96.863%	0.025	97.833%	0.028
bIPCACO-FKNN-V4	98.196%	0.011	97.509%	0.019	99.500%	0.016

Methods	Precision		MCC		F-measure	
	Avg	SD	Avg	SD	Avg	SD
bIPCACO-FKNN-S1	95.785%	0.038	0.84499	0.055	94.126%	0.020
bIPCACO-FKNN-S2	96.763%	0.026	0.87779	0.040	95.384%	0.014
bIPCACO-FKNN-S3	96.763%	0.030	0.87225	0.093	95.266%	0.034
bIPCACO-FKNN-S4	97.742%	0.031	0.87370	0.081	95.300%	0.029
bIPCACO-FKNN-V1	98.710%	0.027	0.94583	0.039	97.910%	0.015
bIPCACO-FKNN-V2	90.946%	0.042	0.72771	0.080	89.796%	0.027
bIPCACO-FKNN-V3	98.710%	0.017	0.94052	0.050	97.767%	0.018

Upon examining the metrics, it is observed that the bIPCACO-FKNN-V4 model achieved the highest values in accuracy, specificity, precision, and F-measure, with respective values of 98.196%, 99.500%, 99.677%, and 98.566%. Particularly noteworthy are its specificity and precision, approaching or achieving 100%, demonstrating a remarkably high capability in identifying non-TSM samples and an extremely low false positive rate.

In contrast, the performance of the bIPCACO-FKNN-V2 model is comparatively inferior, especially in terms of accuracy and specificity, which are only 87.139% and 84.853%, respectively. This suggests that this TF is less effective in distinguishing TSM samples. In terms of MCC and F-measure, the bIPCACO-FKNN-V2 also performs the worst, with values of 0.72771 and 89.796% respectively, reflecting a lower quality and balance in prediction.

The performance of S-shaped TFs (S1 to S4) generally falls between the highest (V4) and lowest (V2) performances of V-shaped TFs. Within this series, the bIPCACO-FKNN-S2 model exhibits better performance, with higher accuracy, sensitivity, and specificity, yet it does not surpass the best model among the V-shaped TFs.

**Figure 14:**
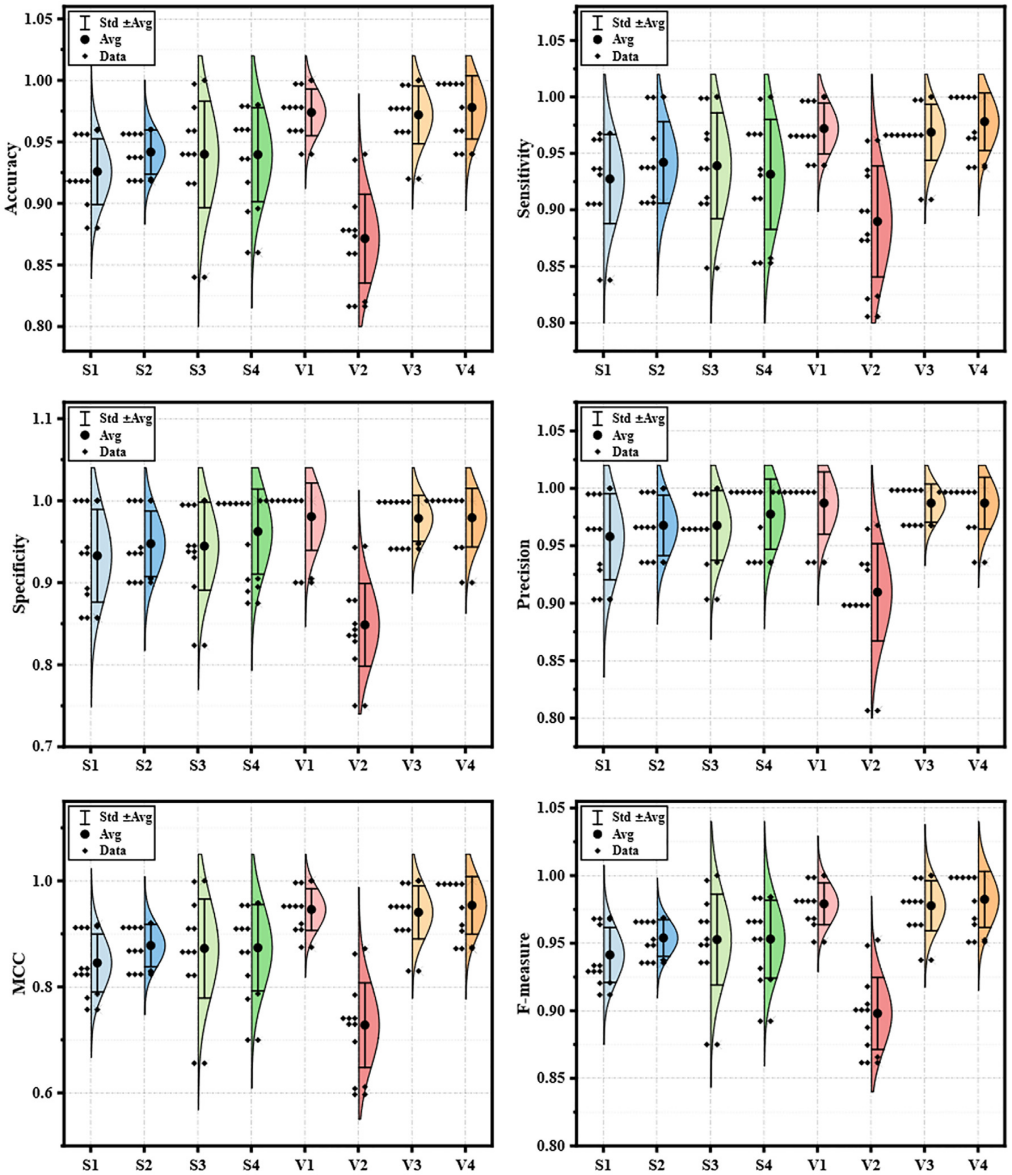
Distribution of evaluation metrics values of bIPCACO-FKNN in predicting TSM on different TFs

Furthermore, Figure 14 shows the distribution of metric values across 10 independent experiments of TSM prediction using bIPCACO-FKNN with different TFs. Comparatively, the S-shaped TF (S2) and V-shaped TFs (V1, V3, and V4), when combined with bIPCACO-FKNN, exhibit relatively higher stability in TSM prediction. S3, S4, and V2, in conjunction with bIPCACO-FKNN, demonstrate lower stability in predicting TSM.

In summary, V-shaped TFs, particularly V4, when integrated with the bIPCACO-FKNN model, provide optimal performance for predicting TSM, showcasing exceedingly high accuracy and diagnostic capability.

### Comparison experiment of predictive models

Following the successful selection of FKNN as the optimal classifier and V4 as the best TF, this study constructed the bIPCACO-FKNN-V4 model, hereafter referred to as bIPCACO-FKNN. To underscore the exceptional performance of the bIPCACO-FKNN model in predicting TSM, it was compared and analyzed against seven FS models based on FKNN and seven well-known classification models. The FS models compared, based on FKNN, included bHHOSRL-FKNN,^59^ bSCGWO-FKNN,^60^ bRFACO-FKNN,^61^ bPSO-FKNN,^62^ bACO-FKNN,^63^ bDE-FKNN,^64^ and bRIME-FKNN.^65^ The selected famous classification algorithms encompassed light gradient boosting machine (LightGBM), extreme gradient boosting (XGBoost), categorical boosting (CatBoost), adaptive boosting (AdaBoost), RandomF, FKNN, and KELM. The parameter settings of these methods are shown in Table 12.

**Table 12:** Parameter settings for the comparison methods

Methods	Parameter values
LightGBM	learning_rate=0.1;Num_Leaves=31
XGBoost	$learning_{rate}=0.3;depth=6$
CatBoost	learning_rate=0.03;depth=6
AdaBoost	NLearn=100
RandomF	NumTrees=20
KELM	k=1;m=2
FKNN	c=88;γ=1024

Table 13 provides the Avg and SD performance metrics of bIPCACO-FKNN, its peer FS models, and well-known classifiers in predicting TSM. Analyzing the data from Table 13, bIPCACO-FKNN demonstrates superior performance across all metrics, particularly in accuracy (98.196%), specificity (99.500%), precision (99.677%), F-measure (98.566%), and MCC (0.96231), markedly outperforming its peers and other well-known classifiers.

**Table 13:** Avg and SD of the bIPCACO-FKNN with its peers and well-known classifiers predicting TSM

Methods	Accuracy		Sensitivity		Specificity	
	Avg	SD	Avg	SD	Avg	SD
bIPCACO-FKNN	98.196%	0.011	97.509%	0.019	99.500%	0.016
bHHOSRL-FKNN	95.188%	0.029	94.886%	0.042	96.274%	0.035
bSCGWO-FKNN	97.196%	0.019	96.893%	0.025	97.892%	0.027
bRFACO-FKNN	96.992%	0.029	96.855%	0.029	97.336%	0.038
bPSO-FKNN	94.588%	0.023	95.063%	0.035	94.390%	0.050
bACO-FKNN	96.400%	0.030	97.264%	0.037	95.602%	0.052
bDE-FKNN	93.571%	0.047	94.098%	0.045	93.182%	0.066
bRIME-FKNN	92.376%	0.024	92.629%	0.035	92.318%	0.036
LightGBM	86.933%	0.032	86.036%	0.038	89.620%	0.063
XGBoost	87.963%	0.053	86.479%	0.051	91.909%	0.079
CatBoost	85.743%	0.049	84.893%	0.059	90.070%	0.085
AdaBoost	82.567%	0.056	81.742%	0.051	85.251%	0.091
RandomF	83.522%	0.054	83.187%	0.046	84.653%	0.085
FKNN	86.539%	0.056	89.136%	0.063	83.533%	0.090
KELM	76.306%	0.049	75.789%	0.051	79.090%	0.085

Methods	Precision		MCC		F-measure	
	Avg	SD	Avg	SD	Avg	SD
bIPCACO-FKNN	99.677%	0.010	0.96231	0.024	98.566%	0.009
bHHOSRL-FKNN	97.731%	0.022	0.89924	0.059	96.221%	0.022
bSCGWO-FKNN	98.710%	0.017	0.94093	0.041	97.772%	0.015
bRFACO-FKNN	98.387%	0.023	0.93652	0.061	97.598%	0.023
bPSO-FKNN	96.441%	0.032	0.88713	0.049	95.675%	0.019
bACO-FKNN	97.097%	0.036	0.92594	0.061	97.103%	0.023
bDE-FKNN	95.796%	0.046	0.86474	0.098	94.874%	0.038
bRIME-FKNN	95.462%	0.023	0.83828	0.054	93.975%	0.019
LightGBM	94.484%	0.035	0.72249	0.072	89.989%	0.024
XGBoost	95.796%	0.043	0.74543	0.115	90.830%	0.040
CatBoost	94.505%	0.059	0.70174	0.103	89.194%	0.035
AdaBoost	92.892%	0.045	0.62592	0.125	86.894%	0.041
RandomF	92.215%	0.044	0.64590	0.119	87.424%	0.041
FKNN	89.613%	0.062	0.71850	0.120	89.192%	0.045
KELM	91.591%	0.041	0.48384	0.116	82.810%	0.032

Compared to other FKNN-based FS models, such as bHHOSRL-FKNN, bSCGWO-FKNN, and bRFACO-FKNN, although these models also showcased commendable performance, they did not match bIPCACO-FKNN in key performance metrics, especially in specificity and precision, further affirming the superiority of bIPCACO-FKNN.

When compared with famous classifiers such as LightGBM, XGBoost, and CatBoost, bIPCACO-FKNN’s performance is significantly superior. Despite the widespread application and generally excellent performance of these classifiers in the field of data science, their key metrics, such as accuracy, sensitivity, and specificity for TSM prediction tasks fall below those of bIPCACO-FKNN, further emphasizing the specific advantages and application value of the bIPCACO-FKNN model.

**Figure 15:**
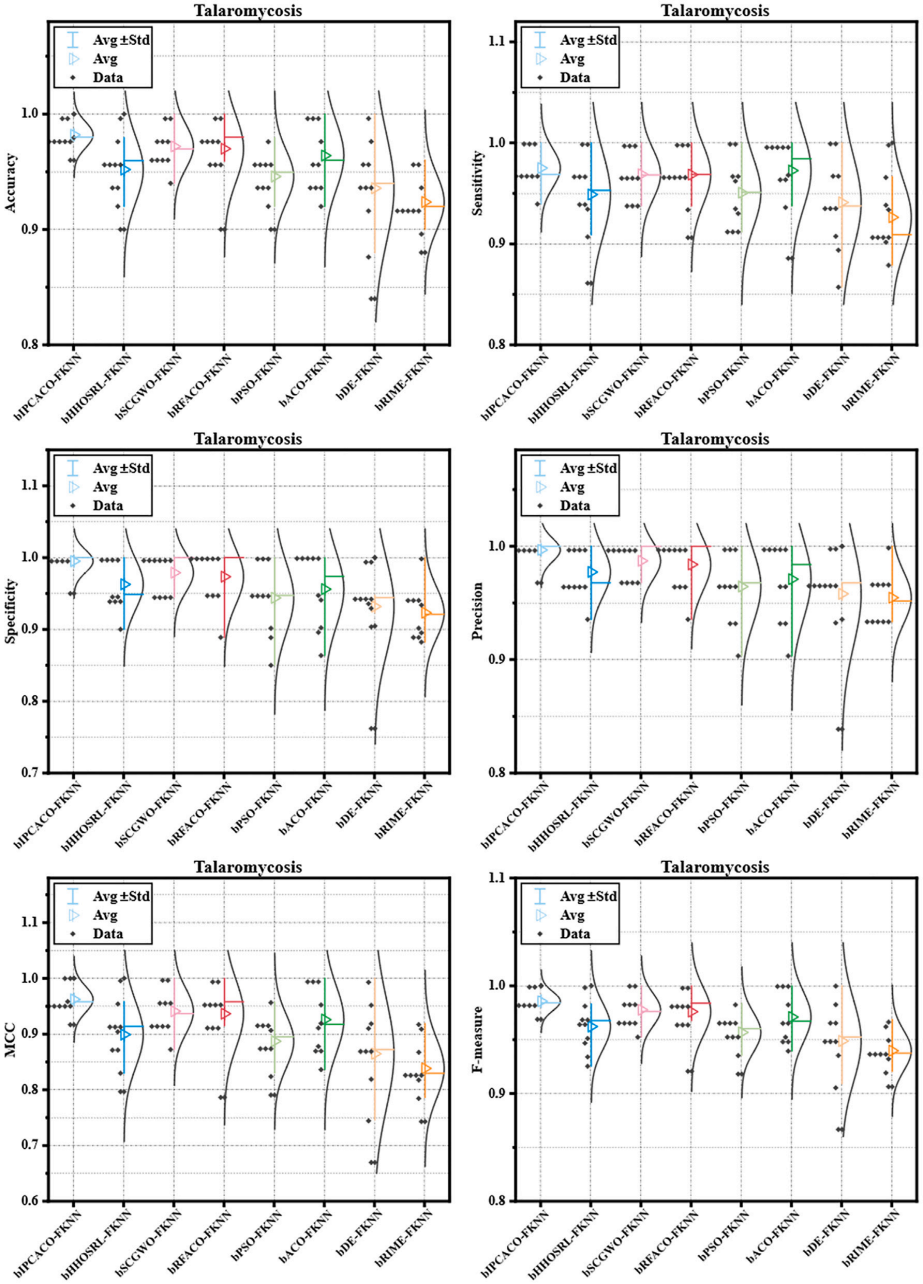
Distribution of metric values for bIPCACO-FKNN versus its peer predicted TSM

**Figure 16:**
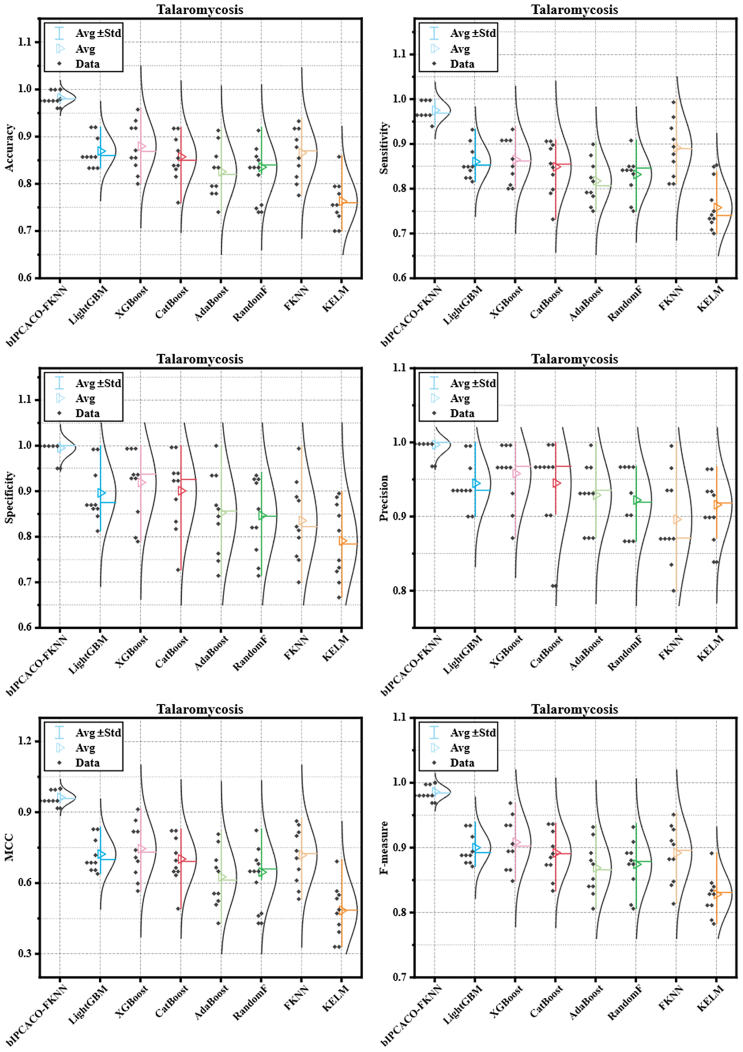
Distribution of metric values for bIPCACO-FKNN versus well-known classifiers predicted TSM

For a more intuitive comparison of different models’ stability in predicting TSM, Figures 15 and 16 depict the performance distribution of competing models across various evaluative metrics. Compared to both its FKNN-based peer FS models and famous classifiers, the bIPCACO-FKNN model demonstrates consistent stability in TSM prediction. Among the FKNN-based peer FS models, bDE-FKNN showed poorer stability in TSM prediction, with RandomF being the lesser among the famous classifiers.

**Figure 17:**
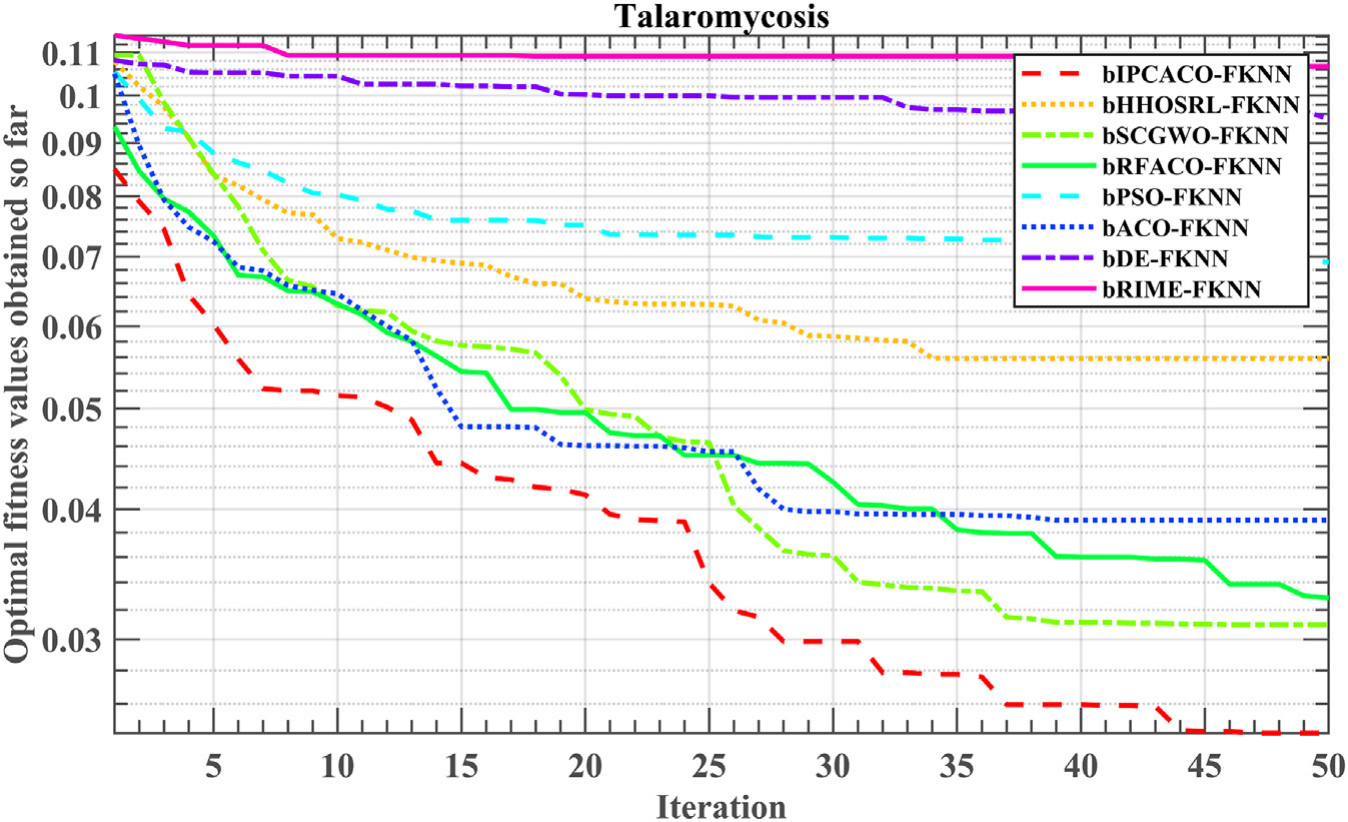
Convergence trend of fitness values of bIPCACO-FKNN with its peers in predicting TSM

Furthermore, Figure 17 provides the convergence trend of peer FS models based on FKNN in identifying the optimal feature subset. Notably, the bIPCACO-FKNN model not only converges fastest but also with the highest precision in identifying the optimal feature subset. Models like bPSO-FKNN, bDE-FKNN, and bRIME-FKNN encountered local optima early on, contributing to their less effective TSM prediction compared to bIPCACO-FKNN.

In conclusion, the bIPCACO-FKNN model exhibits outstanding performance and predictive stability in TSM prediction. Its excellent results across key performance metrics, along with comparative analyses against other FS models and classification algorithms, all attest to the model’s superiority and efficient FS capability. These outcomes underscore the practical application value and potential of the bIPCACO-FKNN model in accurately identifying TSM.

## Discussion

The initial diagnosis of pulmonary TSM is frequently subject to a misdiagnosis and oversight, with tuberculosis being the most common mistaken disease for pulmonary TSM, posing a clinical challenge in terms of imaging differentiation. It is often difficult for clinicians to distinguish between these two diseases by the naked eye alone. We employed an ML approach to conduct feature extraction and image classification for patients with pulmonary TSM and tuberculosis, aiming to assist in the diagnosis of pulmonary TSM. Our findings demonstrated that the proposed bIPCACO-FKNN model achieved an impressive accuracy of 98.196% in distinguishing between pulmonary TSM and pulmonary tuberculosis, with sensitivity and specificity rates reaching 97.509% and 99.500%, respectively. Moreover, the model exhibited high values for MCC (96.231%) and F (98.566%). Based on CT imaging omics, our model effectively discriminates pulmonary TSM from tuberculosis, thereby prompting clinicians to initiate the early diagnosis of TSM for timely disease diagnosis and treatment.

The accuracy, sensitivity and specificity of our ML prediction model for patients with pulmonary TSM are superior to those of conventional diagnostic methods. At present, conventional diagnostic methods for pulmonary TSM include direct microscopy, culture, histopathology, serological examination, and molecular biological detection.^66^ Histopathology and microbial culture serve as the gold standards for diagnosing pulmonary TSM.^67^ However, culture is a time-consuming method with a relatively low positivity rate (only 60%–75% positive rate for blood or bone marrow cultures),^68^ and its sensitivity is significantly lower than that of our machine-predicted model. Invasive procedures are required for histopathology, and clinical specimens are often not readily accessible. Studies have demonstrated that the Mp1p antigen test exhibits exceptional specificity (98.1%), high sensitivity (86.3%), and a shorter diagnostic turnaround time (6 h) than other methods.^69^ However, it should be noted that the sensitivity of this model is lower than that of our machine prediction model. Moreover, there are limited real-world clinical data on the application of Mp1p antigen detection, primarily focusing on HIV-positive patients. Additionally, Gong et al.^70^ reported a decrease in the positivity rate of the Mp1p antigen with an increase in the CD4^+^ T cell count, suggesting a potentially significant reduction in the diagnostic value of the Mp1p antigen for detecting TSM in HIV-negative patients. The mNGS method has been reported to exhibit remarkably high sensitivity (98.3%) and specificity (98.6%) for detecting TSM in HIV-positive patients.^71^ The diagnostic accuracy, sensitivity and specificity of the mNGS method are similar to ours. However, as highlighted in the research conducted by Mao et al., limitations such as high cost and extensive laboratory equipment requirements hinder its widespread clinical implementation.

Currently, there is a lack of imaging omics-based ML models for assisting in the diagnosis of pulmonary TSM, and even AI models for this purpose are rarely reported. Wei et al.^72^ obtained images of skin lesions caused by T. marneffei or Cryptococcus infection from 159 published studies. Subsequently, transfer learning technology was employed to develop five deep AI models—VGG19, MobileNet, InceptionV3, Inception ResNetV2, and DenseNet201—using the image dataset. Among these models, InceptionV3 exhibited superior sensitivity (97.32%), accuracy (96.51%), F value (97.00%), and area under the curve (AUC) value (98.98%). Although this model achieved a commendable diagnostic accuracy, its application scope remains limited because it is suitable only for treating TSMs with skin lesions. Several reports indicate that less than 50% of HIV-negative TSMs exhibit such lesions.^73^ Using logistic regression and random forest analysis, Hu et al.^74^ constructed a random forest model for diagnosing TSM. However, its sensitivity was comparable to that of microbial culture, ranging from 78.2% to 83.2%. Additionally, the area under the receiver operating characteristic (ROC) curve was determined to be 0.859, indicating that the proposed model is less effective than our constructed diagnostic model.

The AI diagnostic model constructed by our ML method based on imaging features has demonstrated comparable or superior diagnostic sensitivity, specificity, and accuracy (up to 98.196%) when compared to existing conventional diagnostic methods and AI models. Moreover, it offers the advantages of simplicity, rapid prediction, and cost-effectiveness. This model does not necessitate invasive procedures or even basic blood tests, rendering it highly suitable for elderly or critically ill patients who may have difficulty tolerating invasive techniques such as tracheoscopy and lung puncture. Moreover, the model relies solely on chest CT images without the need to incorporate other blood test results, resulting in a relatively low cost and enabling its utilization in economically underdeveloped regions. But it cannot completely replace traditional methods. Culture and histopathology remain the gold standard for the diagnosis of TSM. The success of the ML model allows doctors to quickly consider the disease in the first time, start the diagnosis process as soon as possible, and avoid misdiagnosis as pulmonary tuberculosis or other pulmonary diseases. The ML model is made into an application software, which is integrated into the doctor’s tablet or computer to quickly identify the characteristics of HIV-negative TSM, start the diagnosis process, and confirm the diagnosis of TSM.

However, our model also has certain limitations. First, in terms of the research scope, we focused solely on distinguishing pulmonary TSM from tuberculosis without considering other pulmonary diseases. This may somewhat affect the diagnostic applicability of our model. Nevertheless, in terms of imaging, pulmonary tuberculosis is the pulmonary disease most prone to confusion with pulmonary TSM and has the highest rate of misdiagnosis. This phenomenon has been extensively reported in numerous studies. The distinction between pulmonary tuberculosis and pulmonary TSM by imaging poses a significant challenge, but the model we developed will greatly assist clinicians in this respect. Second, this study included a limited number of patients from a single hospital and lacked external validation datasets, thus hindering the widespread application of this model. Nonetheless, internally validated results have consistently demonstrated good discrimination ability throughout the exploration process. In the future, we will increase both the sample size and the diversity of pulmonary disease reports and conduct multicenter studies to further enhance and optimize the model’s applicability and stability for improved implementation in clinical practice.

### Limitations of the study

However, there are also some limitations. It should be noted that the FS method proposed in this study is based on wrapper-based FS. Therefore, when dealing with high-dimensional data, the proposed method may not balance the quantity and precision of the selected feature subset. As a result, future studies might investigate applying the proposed algorithm to various other feature selection methods. Additionally, incorporating the PID strategy has led to an increase in the algorithm’s time complexity. Thus, future efforts will focus on designing effective strategies that can improve algorithm performance while reducing time overhead. In addition, different groups may have different symptoms of the disease, and these differences may affect the accuracy and universality of the prediction model. Therefore, in the future, more attention can be paid to the impact of different group characteristics on model performance to ensure that the model can be more widely and accurately applied to various populations.

## Resource availability

### Lead contact

Further information and requests for resources and reagents should be directed to and will be fulfilled by the lead contact, Huiling Chen (chenhuiling.jlu@gmail.com).

## Materials availability

This study did not generate new unique reagents.

### Data and code availability

• The talaromycosis dataset used in this article can be found in the key resources table.
• All original code has been deposited at Github at https://github.com/2023Wcv/ML and is publicly available as of the date of publication.
• Any additional information required to reanalyze the data reported in this paper is available from the lead contact upon request.

## Acknowledgments

This work was supported by the Project of 10.13039/501100007194Wenzhou Science and Technology Bureau (grant number Y20240209).

## Author contributions

Y.Z., writing—original draft, writing—review and editing, software, visualization, and investigation; P.L., writing—original draft, writing—review and editing, software, visualization, investigation, conceptualization, methodology, and formal analysis; L.X., writing—original draft, writing—review and editing, software, visualization, and investigation; A.S.H., writing—review and editing, software, visualization, and investigation; Y.C., writing—original draft, writing—review and editing, software, visualization, and investigation; L.L., writing—original draft, writing—review and editing, software, visualization, and investigation; H.C., conceptualization, methodology, formal analysis, investigation, writing—review and editing, funding acquisition, supervision, and project administration; C.L., conceptualization, methodology, formal analysis, investigation, writing—review and editing, funding acquisition, supervision, and project administration; Y.L., conceptualization, methodology, formal analysis, investigation, writing—review and editing, funding acquisition, supervision, and project administration.

## Declaration of interests

The authors declare no competing interests.

## Declaration of generative AI and AI-assisted technologies

During the revision stage of this work the author(s) used ChatGPT as a grammar checker in order to double check and proofread the English grammar of the paper. After using this tool/service, the author(s) reviewed and edited the content as needed and take(s) full responsibility for the content of the publication.

## STAR★Methods

### Key resources table

**Table:** 

REAGENT or RESOURCE	SOURCE	IDENTIFIER
**Deposited data**		

The talaromycosis dataset	This paper	https://github.com/2023Wcv/ML
Code for method	This paper	https://github.com/2023Wcv/ML

**Software and algorithms**		

MATLAB R2022b	Mathworks	www.mathworks.com

### Experimental model and study participant details

#### Study participant details

##### Patient population

The present study adhered to the principles outlined in the Declaration of Helsinki and complied with relevant ethical requirements, receiving approval from the Institutional Review Committee of the First Affiliated Hospital of Wenzhou Medical University. A retrospective study was conducted on patients with confirmed pulmonary TSM admitted to the First Affiliated Hospital of Wenzhou Medical University from January 1, 2014 to June 30, 2023. HIV-positive patients or those without pulmonary involvement were excluded, resulting in a total of 48 patients with HIV-negative pulmonary TSM. Propensity matching at a ratio of 1:2 was performed to screen for age- and sex-matched HIV-negative pulmonary tuberculosis patients during the same period, and 96 patients were ultimately enrolled. Patient data, including age, sex, underlying disease status (such as hypertension, diabetes mellitus, solid organ tumors, and so on), microbiological detection results of clinical specimens (sputum, alveolar lavage fluid, blood culture, lung puncture tissue culture, TB-Xpert MTB/RIF) and chest CT scans, were retrospectively collected through the electronic medical records system. Informed consent was waived because this was a retrospective study, and there was no modification in patient management.

##### Diagnostic criteria

Pulmonary tuberculosis^75^: Mycobacterium tuberculosis was isolated from sputum, alveolar lavage fluid or lung puncture tissue culture, or the presence of Mycobacterium tuberculosis was confirmed by TB-Xpert MTB/RIF or lung histopathology.

TSM^76^:T. marneffei was isolated from a blood culture or other clinical specimens, or the presence of T. marneffei infection was confirmed via histopathology of biopsy specimens.

### Method details

#### Fuzzy k-nearest neighbors

FKNN^77^ is an improved classifier that introduces fuzzy concepts into the decision rules of the classic KNN. Fuzzy sets express the uncertainty of sample membership degrees for each class label. FKNN learns from fuzzy sets to generate fuzzy classification rules. Similar to KNN, FKNN selects the K nearest neighbors for a given sample $x_{i}$. Subsequently, the membership count for each class l is calculated using Equation 1.

(Equation 1) $$\mu \left(x,l\right)=\frac{\sum_{j=1}^{K}\mu \left(x_{j},l\right)\left(1/{\left\|x-x_{j}\right\|}^{2/\left(m-1\right)}\right)}{\sum_{j=1}^{K}\left(1/{\left\|x-x_{j}\right\|}^{2/\left(m-1\right)}\right)}$$

where the parameter m determines the extent of influence of neighbor distances. For sample $x_{i}$, the label l with the maximum membership degree will be assigned as the label for the given sample.

#### Overview of the ACO algorithm

The ACO algorithm,^27^ inspired by the foraging behavior of ants in nature, constitutes a Mas. In the natural world, ants locate food sources through the secretion and detection of pheromones, laying down pheromone trails as they return to their nests, thereby guiding other ants to the food source. Drawing on this mechanism, the ACO algorithm is designed to tackle a variety of combinatorial optimization problems. It simulates the exchange of information and collaboration among ant colonies to effectively search for optimal solutions within a search space.

The quintessence of the ACO algorithm lies in its emulation of ants' pheromone release and detection during the search process, thereby directing the algorithm’s search trajectory. Initially, each ant selects a path at random, determining its direction and distance based on pheromone concentration and heuristic information as the search progresses. Upon discovering a solution, an ant will release pheromones along its return path, with the quantity of pheromones directly proportional to the quality of the solution. Consequently, other ants are guided by the pheromones during their search, favoring paths with higher pheromone concentrations. This, in turn, facilitates the iterative optimization of solutions.

As research has advanced, the ACO algorithm has undergone continuous development and refinement. In 2008, Socha et al.^63^ introduced the Ant Colony Optimization for Continuous domains (ACOR) algorithm, extending the applicability of the ACO algorithm from traditional discrete optimization problems to continuous optimization problems. The ACOR algorithm encompasses three critical stages: pheromone initialization, sampling operations, and pheromone update, each of which will be described in detail subsequently.

##### Pheromone initialization

During the initialization phase of the ACOR algorithm, m ants are randomly distributed within the search space, marking the commencement of the algorithm’s solution-seeking process. Subsequently, as the algorithm runs, each ant assumes the role of a search agent, adjusting its position within the search space based on pheromone information collected from the environment. These pheromones, deposited by ants upon discovering efficacious paths or solutions, serve to guide subsequent ants toward more promising regions for exploration.

To ensure that pheromone information effectively directs ants toward optimal solutions, the algorithm incorporates a solution archiving mechanism. The essence of this mechanism lies in the establishment of a repository specifically designated for the preservation of a collection of high-quality solutions. This repository selects and retains k representative efficient solutions, with each solution $s_{l}=\left\{s_{l}^{1},s_{l}^{2},\cdots ,s_{l}^{n}\right\}$ and its corresponding fitness value $f\left(s_{l}\right)$ recorded and stored. Through such an arrangement, ants are not only able to learn from the current distribution of pheromones but also gain invaluable insights from these historical high-quality solutions, thereby further enhancing the directionality and efficiency of the search process. Figure S1 provides a schematic illustration of the configuration of this solution repository, offering a visual representation of how these high-quality solutions are organized and utilized.

The k solutions contained within the archive are ranked according to their fitness values. This ranking ensures that high-quality solutions are given priority, thereby directing ants toward more promising regions for exploration. When condition $f\left(s_{1}\right)\leq \ldots f\left(s_{l}\right)\leq \ldots f\left(s_{k}\right)$ occurs, it precipitates event $w_{1}\geq \ldots w_{l}\geq \ldots w_{k}$. Furthermore, each solution in the archive, such as $s_{l}$, is assigned a weight $w_{l}$. This weight $w_{l}$ plays a pivotal role within the algorithm, as it determines the probability $p_{l}$ of the solution $s_{l}$ being selected as a reference entity. In other words, the weight $w_{l}$ directly influences the significance of the solution $s_{l}$ in the subsequent search process. Ants, when choosing their next direction of exploration, make decisions based on these weights, thereby optimizing the search trajectory. The formula for calculating the probability $p_{l}$ is as follows:

(Equation 2) $$w_{l}=\frac{1}{qk\sqrt{2\pi }}e^{-\frac{{\left(l-1\right)}^{2}}{2q^{2}k^{2}}}$$

(Equation 3) $$p_{l}=\frac{\omega_{l}}{\sum_{r=1}^{k}\omega_{r}}$$

where k represents the capacity of the archive bag used for holding the ACOR’s pheromone, and q is a constant that balances the ACOR algorithm’s local exploitation with its global exploration.

##### Sampling operations

Sampling operations constitute a critical phase within the ACOR algorithm, tasked with generating new solutions based on the current pheromone distribution. The essence of this process lies in employing probability density functions for sampling, thereby exploring the solution search space. However, traditional probability density functions often adopt unimodal Gaussian functions. While this functional form is notably effective in addressing simple problems, its performance is limited when confronting complex distributions characterized by multiple peaks. This limitation stems from the inability of a single Gaussian distribution to accurately capture the distribution characteristics surrounding each local optimum in multimodal problems, thereby impeding the algorithm’s effectiveness and efficiency in identifying global optima.

To surmount this limitation and enhance the algorithm’s capability to tackle multimodal problems, ACOR introduces a mechanism based on Gaussian kernel functions. This mechanism constructs a more complex probability density function capable of effectively approximating multimodal distributions by linearly weighting and summing multiple one-dimensional Gaussian functions. The mathematical representation of this approach is as follows:

(Equation 4) $$G^{i}\left(x\right)=\sum_{l=1}^{k}w_{l}g_{l}^{i}\left(x\right)$$

(Equation 5) $$g_{l}^{i}\left(x\right)=\frac{1}{\sigma_{l}^{i}\sqrt{2\pi }}e^{-\frac{{\left(x-\mu_{l}^{i}\right)}^{2}}{2{\sigma_{l}^{i}}^{2}}}$$

(Equation 6) $$\sigma_{l}^{i}=\xi \sum_{r=1}^{k}\frac{\left|s_{r}^{i}-s_{l}^{i}\right|}{k-1},\xi >0$$

where the sampling operations involve three crucial coefficient vectors: the weight vector $w=\left\{w_{1},w_{2},\ldots ,w_{k}\right\}$, the mean vector $\mu^{i}=\left\{\mu_{1}^{i},u_{2}^{i},\ldots ,\mu_{k}^{i}\right\}$, and the standard deviation vector $\sigma^{i}=\left\{\sigma_{1}^{i},\sigma_{2}^{i},\ldots ,\sigma_{k}^{i}\right\}$. The determination of the mean vector $\mu^{i}$ depends on the selection of sampled individuals affected by the probability $p_{i}$. Moreover, the parameter ξ signifies the evaporation rate of the pheromone.

##### Pheromone update

During the execution of the ACOR algorithm, the pheromone update step following sampling operations is pivotal, exerting a direct influence on the algorithm’s search direction and efficiency. Specifically, the distribution of pheromones is adjusted and updated based on the quality of solutions obtained at the current juncture, thereby guiding the algorithm more effectively toward the global optimum.

During the execution of the ACOR algorithm, the pheromone update step following sampling operations is pivotal, exerting a direct influence on the algorithm’s search direction and efficiency. Specifically, the distribution of pheromones is adjusted and updated based on the quality of solutions obtained at the current juncture, thereby guiding the algorithm more effectively toward the global optimum.

Upon executing sampling operations and acquiring new solutions, the algorithm initially merges these newly obtained m solutions with the k high-quality solutions already present in the solution archive. Consequently, a total of m+k candidate solutions are amassed. Subsequently, the algorithm evaluates and ranks these m+k solutions according to their fitness values. Solutions with higher fitness values are indicative of closer proximity to the problem’s optimal solution, thus holding greater value. Based on this assessment, the algorithm selects the top k solutions with the highest fitness values as the new sources of pheromones. These selected solutions are utilized to update the pheromone distribution, reflecting the best outcomes obtained in the most recent search activities. The remaining m solutions, due to their relatively lower quality, are no longer retained in the solution archive.

#### Proposed IPCACO algorithm

##### Incremental PID control search

The IPC strategy originates from the PID-based search algorithm,^78^ employing the PID regulatory mechanism to guide the search process toward identifying the global optimum solution. Specifically, the algorithm dynamically adjusts the positions of the search agents (individuals) in response to the deviation from the target solution. The PID regulation component within this strategy encompasses a detailed mathematical model and procedural description, segmented into four phases: calculating system deviations, PID regulation, zero output adjustment, and position updating. The pseudo-code for the IPC strategy is shown in Algorithm 1.

Algorithm 1 **Incremental PID control search (IPC)**

Input: X(t),Fit,k,FEs,MaxFEs.

Output: an optimized population X and FEs.

1. Parameter settings including $K_{p},K_{i}$ and $K_{d}$;

2. Updates the deviations $e_{k}\left(t\right)$, $e_{k-1}\left(t\right)$, and $e_{k-2}\left(t\right)$ by Equations 7, 8, and 9;

3. Calculate Δu(t) based on $e_{k}\left(t\right)$, $e_{k-1}\left(t\right)$, and $e_{k-2}\left(t\right)$ by Equation 10;

4. Calculate the zero output adjustment term o(t) by Equations 11, 12, 13, and 14;

5. Updates the $X_{PID}$ based on X(t), Δu(t), and o(t) by Equations 15 and 16;

6. for i=1 to k do

7.  if $f\;\left(X_{PID}\left(i\right)\right)<{Fit}_{i}$

8. ${\;X}_{i}\left(t+1\right)\leftarrow X_{PID}\left(i\right)$;

9.  ${Fit}_{i}\leftarrow f\;\left(X_{PID}\left(i\right)\right)$;

10.  end if

11.  FEs←FEs+1;

12. end for

###### Calculating system deviations

In order to incorporate historical states and furnish the PID regulation with a more comprehensive dataset, the IPC strategy necessitates the calculation of not only the current deviation, denoted as $e_{k}\left(t\right)$, but also $e_{k-1}\left(t\right)$ and $e_{k-2}\left(t\right)$, which represent the deviations from the previous and the penultimate iterations, respectively. The formulation of these calculations is presented as follows:

(Equation 7) $$e_{k}\left(t\right)=X_{best}\left(t-1\right)-X\left(t-1\right)$$

(Equation 8) $$e_{k-1}\left(t\right)=e_{k}\left(t-1\right)+X_{best}\left(t\right)-X_{best}\left(t-1\right)$$

(Equation 9) $$e_{k-2}\left(t\right)=e_{k-1}\left(t-1\right)$$

wherein, at the inception of the process, specifically at t=1, the $e_{k}\left(t\right)$, $e_{k-1}\left(t\right)$, and $e_{k-2}\left(t\right)$ are set to equal values, thus establishing the initial point for the algorithm’s iterative sequence. As the algorithm progresses, these deviation values are dynamically updated in accordance with the actual conditions encountered. The notations $X_{best}\left(t\right)$ and $X_{best}\left(t-1\right)$ respectively represent the positions of the global optimum solution at the tth and (t−1)th iterations. The X(t−1) denotes the position of the current individual at the iteration (t−1)th. The deviation $e_{k-1}\left(t\right)$ calculated in the previous generation, and the difference $X_{best}\left(t\right)-X_{best}\left(t-1\right)$ mirrors the change in the global optimum solution across two consecutive generations, thereby facilitating the dynamic adjustment of the deviation values.

###### PID regulation

Based upon the deviations $e_{k}\left(t\right)$, $e_{k-1}\left(t\right)$, and $e_{k-2}\left(t\right)$, the adjustment in position for each individual, denoted as Δu(t), is determined through the following PID formula:

(Equation 10) $$\Delta u\left(t\right)=K_{p}\cdot r_{1}\cdot \left(e_{k}\left(t\right)-e_{k-1}\left(t\right)\right)+K_{i}\cdot r_{2}\cdot e_{k}\left(t\right)+K_{d}\cdot r_{3}\cdot \left(e_{k}\left(t\right)-{2e}_{k-1}\left(t\right)+e_{k-2}\left(t\right)\right)$$

where $K_{p}$, $K_{i}$, and $K_{d}$ represent the proportional, integral, and differential coefficients within the PID control, respectively, these coefficients are set to values of 1, 0.5, and 1.2, correspondingly. The variables $r_{1}$, $r_{2}$, and $r_{3}$ are vectors of random numbers within the interval [0,1], introduced to incorporate randomness into the process.

###### Zero output adjustment

To augment the global search capability of the IPC strategy and prevent premature convergence, a zero output adjustment term, o(t), is introduced. The objective of this term is to offer an additional dynamic exploration mechanism beyond the PID regulation. The specific computational formula is as follows:

(Equation 11) $$o\left(t\right)=\left(\cos\left(1-\frac{FEs}{MaxFEs}\right)+\lambda \cdot r_{4}\cdot Levy\right)\cdot e_{k}\left(t\right)$$

(Equation 12) $$\lambda ={\left(\frac{\ln\left(MaxFEs-FEs+2\right)}{\ln\;MaxFEs}\right)}^{2}$$

(Equation 13) $$Levy\approx \frac{u}{{\left|v\right|}^{1/\beta }},u∼N\left(0,\sigma_{u}^{2}\right),v∼N\left(0,\sigma_{v}^{2}\right),\sigma_{v}=1,\beta =3/2$$

(Equation 14) $$\sigma_{u}={\left\{\frac{\Gamma \left(1+\beta \right)\cdot \sin\left(\pi \cdot \beta /2\right)}{\Gamma \left[\left(1+\beta \right)/2\right]\cdot \beta \cdot 2^{\left(\beta -1\right)/2}}\right\}}^{1/\beta }$$

where FEs represents the current number of evaluations, and MaxFEs denotes the maximum number of evaluations allowable. The parameter λ is an adjustment coefficient used to control the intensity of the influence exerted by the Lévy flight term. The variable $r_{4}$ is a vector of random numbers within the range [0,1], introduced to enhance randomness and facilitate the exploration of new solution spaces.

###### Position updating

The new position of an individual, X(t+1), is ascertained by amalgamating the PID regulation value with an additional adjustment term o(t), with the update formula being expressed as follows:

(Equation 15) X(t+1)=X(t)+η·Δu(t)+(1−η)·o(t)

(Equation 16) $$\eta =r_{5}\cdot \cos\;FEs/MaxFEs$$

where η is a dynamic weight employed to balance the contributions of the PID regulation term Δu(t) and the additional adjustment term o(t) to the update of the position.

#### IPCACO algorithm

In the ACO algorithm, a pivotal mechanism involves guiding the search behavior of ants through the positive feedback of pheromones. This mechanism enables the ants to identify high-quality paths within the solution space. However, this approach has its limitations: within each generation, only the pheromones of the first k optimal solutions are preserved and reinforced, often neglecting the actual efficacy and diversity of these solutions. Reliance solely on the pheromone updates of local optima may result in the search process becoming ensnared in local optima, making it challenging to escape. To enhance the quality of the pheromone solutions, an improved ACO algorithm incorporating an IPC strategy, termed IPCACO, has been proposed. Through the IPC strategy, the algorithm dynamically adjusts the pheromone update strategy, considering not only the currently identified optimal solutions but also adapting the search strategy in response to changes encountered during the search process, thereby maintaining the flexibility and efficiency of the search.

Algorithm 2 delineates the pseudocode for the IPCACO algorithm, which, by integrating the IPC strategy, augments the search efficiency and quality of solutions. Initially, the algorithm is initialized through parameter settings and establishes an archive sorted by fitness values. At the start of each iteration within the main loop, a new ant population is initialized. For each ant in the population, the algorithm probabilistically selects a guiding solution and generates the ant’s solution by Equations 4, 5, and 6, subsequently calculating its fitness value. The solutions generated by all ants are then merged with those in the archive, sorted by fitness values, and the optimal solutions are placed into the archive. Furthermore, the IPC strategy is employed to dynamically adjust the search process, thus enhancing the quality of the pheromone solutions. This process continues until the maximum number of evaluations is reached. At that point, the currently identified optimal solution and its fitness value are outputted.

Algorithm 2 **IPCACO framework**

**Input:**D,m,ub,lb,MaxFEs.

**Output:** optimal individual $X_{best\;}$ and its fitness value $f\;\left(X_{best}\right)$.

1. Parameter settings including k,FEs;

2. Initialize the archive $S=\left\{s_{1},s_{2},\ldots ,s_{k}\right\}$ and sort them according to their fitness values Fit;

3. FEs←FEs+k;

**4. while** FEs≤MaxFEs **do**

5.  Initialize a population $X=\left\{X_{1},X_{2},\ldots ,X_{m}\right\}$;

**6.** **for**i=1 to m **do**

7.  Determining the guiding solution $s_{l}$ based on the probability $p_{l}$;

8.  Generate the ith ant $X_{i}$ through Equations 4, 5, and 6;

9.  Calculating the fitness value $f\;\left(X_{i}\right)$ of member $X_{i}$;

10.  FEs←FEs+1;

11.  end for

12.  Sorting is performed on the combined k+m ants according to their fitness values;

13.  Placing the top k ants of better quality into the archive bag in the sequence is performed;

14.  (X,FEs)← Incremental PID control search (X,Fit,k,FEs,MaxFEs);

15.  Update to the current position of the $X_{best\;}$ and its fitness value $f\;\left(X_{best}\right)$;

16. end while

#### Computational complexity of IPCACO

Time complexity is a crucial metric for evaluating the efficiency of an algorithm.^79^^,^^80^ An analysis of the pseudocode described in Algorithm 2 reveals that the algorithm encompasses initialization, a cyclic search process, and the update process for the IPC. The complexity analysis for each stage is as follows:

##### Parameter setting and initialization

This stage primarily involves the preparatory tasks of the algorithm, including the setting of parameters and the initialization of the solution archive. The complexity of this process generally relates to the initial size of the archive. Assuming the archive size is k and the problem dimension is D, the complexity of this stage is approximately O(k·logk+k·D), as it necessitates sorting the initial solution archive.

##### Cyclic search process

The core of the algorithm is a cyclic process, with the number of cycles depending on the termination condition, assumed to be MaxFEs. Each cycle executes the following key steps:

(1) Initialization of the population and generation of new solutions: The complexity of this step depends on the population size m, the problem dimension D, and the archive size k, yielding a complexity of approximately kO(m·D·).
(2) Pheromone update: Updating the pheromones involves sorting and selecting operations for m+k solutions, with a complexity of O(k+m)·log(k+m)).
(3) IPC strategy: This step involves adjusting the search process based on the IPC strategy. Its complexity is O(k·D+k).

Combining the above steps, the overall computational complexity of the IPCACO algorithm can be roughly estimated as O(k·log·k+k·D+MaxFEs·((k+m)·log(k+m)+k·D·(m+1)+k)). The complexity of the algorithm is influenced by the maximum number of evaluations MaxFEs, the size of the ant colony m, the problem dimension D, and the size of the solution archive k.

##### Proposed bIPCACO-FKNN model

Figure S2 depicts the flowchart for developing a wrapper-based FS model using IPCACO for feature subset selection and FKNN as the classifier. To improve the model’s generalization capability, 10-fold cross-validation is applied during model training. In this method, the dataset is divided into ten parts, with nine parts used for training and one part reserved for validation. This process is repeated ten times to ensure that each sample is included in both the training and testing phases of the model, thereby providing a comprehensive evaluation of its performance.

For a given TSM image, three methods (color matrixes, gray-level co-occurrence matrix (GLCM), and local binary patterns (LBPs)) are first employed to extract image features.

The theory of color matrixes posits that all colors in an image can be represented in matrix form. The first and second moments are used to describe the central tendency and dispersion of color distribution, respectively. In TSM images, color matrixes can extract statistical characteristics of each channel, thereby capturing the overall color properties of the image. The GLCM is a statistical method for texture feature extraction, describing texture information by analyzing the joint probability distribution of pixel intensity values at specific orientations and distances. GLCM can compute various texture features, such as contrast, entropy, correlation, and energy, enabling precise capture of the microstructural texture of the image. When applied to TSM images, GLCM reflects both local and global texture characteristics. The LBP method, on the other hand, compares the intensity value of each pixel with that of its neighboring pixels, encoding the results into binary patterns. This approach effectively captures local texture patterns in the image, such as edges, corners, and uniform regions.

After completing the feature extraction, a 10-fold cross-validation method is used to evaluate model performance and mitigate the risk of overfitting. The dataset is randomly partitioned into ten subsets of approximately equal size, with one subset selected as the test set and the remaining nine subsets used as the training set in each iteration. When training the model using the training set, IPCACO serves as the feature subset search method to search for the optimal feature subset. It is important to note that the search space for FS is a discrete space represented by ‘0’ and ‘1’, where ‘0’ indicates the feature is not selected and ‘1’ indicates the feature is selected. However, IPCACO proposed in Section 3 is designed for continuous problems. Therefore, it is necessary to transform the search individual positions from continuous space to discrete space using a transfer function (TF). There are commonly eight types of TFs, including S-shaped and V-shaped TFs. Based on experiments (the results are presented in Section 6.2), this study selects the following TF for transforming the search individual positions:

(Equation 17) $$V\left(x\right)=\left|\frac{2}{\pi }\times \tan^{-1}\left(\frac{\pi }{2}\times x\right)\right|$$

(Equation 18) $$X_{i}\left(t\right)=\left\{ \begin{array}{cc} {∼X}_{i}\left(t\right), & V\left(X_{i}\left(t\right)\right)>rand\\ X_{i}\left(t\right), & \text{otherwise}\; \end{array}\right.$$

where rand is a random number range from 0 to 1.

Furthermore, when using binary IPCACO (bIPCACO) to search for feature subsets, it is necessary to use a fitness function to evaluate the quality of the current feature subset. In FS, the number of features in the subset and the accuracy of the classification model are two primary considerations. Therefore, based on these two aspects, this study employs the following fitness function for evaluating the quality of feature subsets:

(Equation 19) $$f\left(x\right)=\alpha \times \left(1-{Acc}_{FKNN}\right)+\rho \times \frac{Number\;of\;selected\;features}{Total\;features}$$

where ${ACC}_{FKNN}$ represents the classification accuracy of the FKNN classifier. α and ρ are two control parameters with values set to 0.99 and 0.01, respectively.

When the bIPCACO algorithm reaches the maximum number of iterations, the optimal feature subset is output. Based on this optimal feature subset, an FKNN model is constructed and its performance is evaluated on the test set by calculating classification performance metrics for a single test. The above process is repeated ten times, with each iteration conducted on a different test set partition. By averaging the results from multiple repetitions, the final average classification performance of the model is obtained.

### Quantification and statistical analysis

The statistical analysis was conducted using SPSS 20 software (SPSS, Inc., Chicago, IL, USA). Continuous variables are expressed as medians (25th, 75th percentiles) or means ± standard deviations (SDs), depending on whether they followed a normal distribution. Categorical variables are presented as counts (percentages). To compare differences in baseline characteristics, Student’s t test and Mann-Whitney U test were employed as appropriate, along with the chi-square test. When the application conditions were not met, the continuity correction χ2 test or Fisher’s exact test was used. All tests were two-tailed, with a *p* value ≤0.05 indicating statistical significance.

A total of 144 patients were enrolled in this study, and the mean age of the patients in both groups was 56.5 ± 15.8 years. There were 48 patients in the TSM group: 40 males and 8 females. Among these patients, 45 had an underlying disease, predominantly hypertension, diabetes, liver disease, or renal insufficiency. In the pulmonary tuberculosis group, there were a total of 96 patients—80 males and 16 females. Among these patients, 59 had underlying diseases, primarily hypertension and diabetes. In total, the study included 497 CT scan images, comprising 189 images from TSM cases and 308 images from Non-TSM cases. Regarding underlying disease, the proportions of patients who underwent solid organ transplantation, had malignant tumors, and had immune-related diseases were significantly greater in the TSM group than in the tuberculosis group (*p* < 0.05). The details are shown in Table S5.
