# Supplementary material for: An enhancing diagnostic pulmonary diseases diagnostic method for differentiating talaromycosis from tuberculosis
Source: iScience. 2025 Jan 22;28(2):111867. doi: 10.1016/j.isci.2025.111867 (PMC11872622; doi:10.1016/j.isci.2025.111867)
Supplement: Document S1. Figures S1 and S2, Tables S1–S4 [file mmc1.pdf]

iScience, Volume *28*

Supplemental information

An enhancing diagnostic pulmonary diseases diagnostic method for differentiating talaromycosis from tuberculosis

Ying Zhou, Pengchen Lin, Lijing Xia, Ali Asghar Heidari, Yi Chen, Lei Liu, Huiling Chen, Chengye Li, and Yuping Li

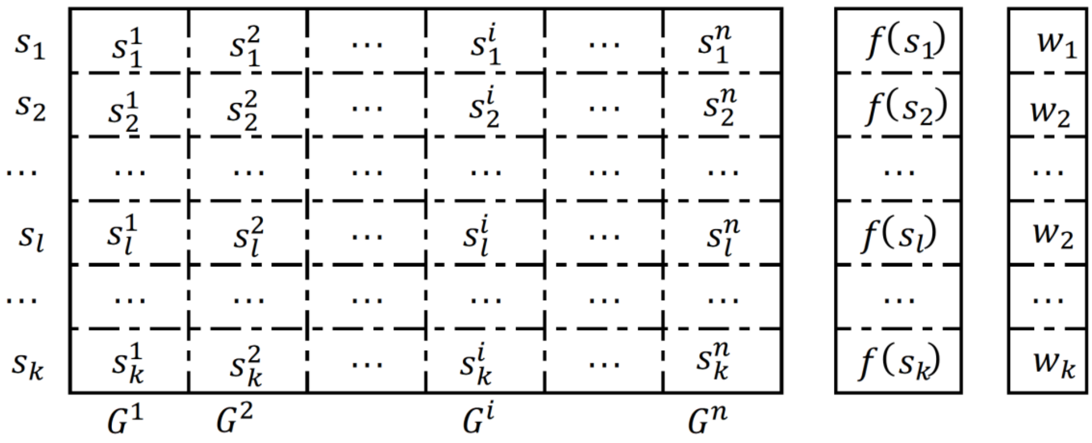

Figure S1. The archive with k ants kept in ACOR.

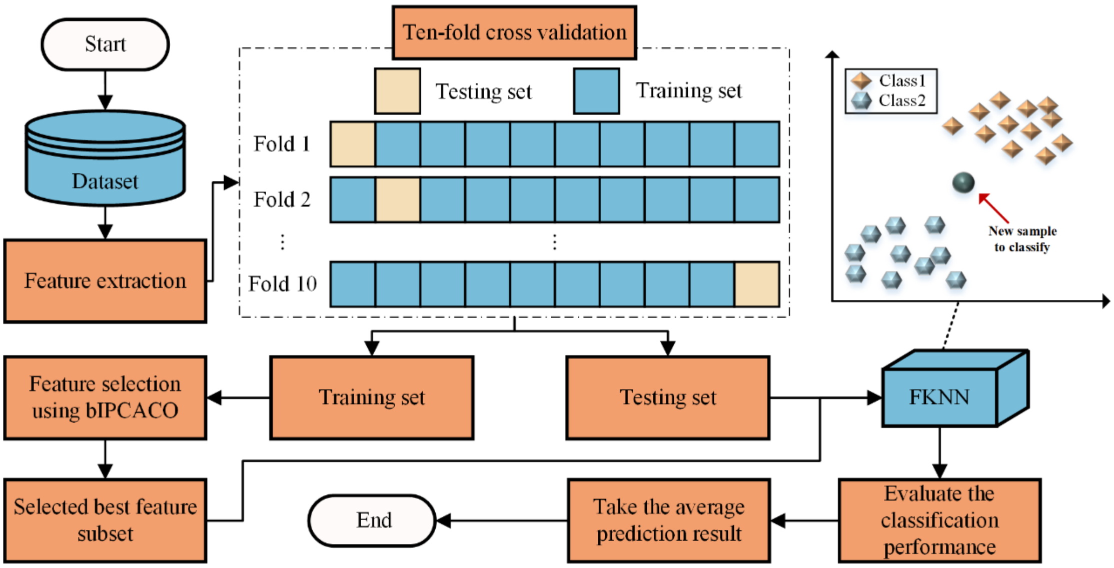

Figure S2. Flowchart of bIPCACO-FKNN model.

Table S1. Avg and Std results of IPCACO and comparative algorithms.

	F1		F2		F3	
	Avg	Std	Avg	Std	Avg	Std
IPCACO	3.36558E+03	4.16396E+03	**3.00000E+02**	**4.45725E-10**	**4.37740E+02**	**3.24060E+01**
ACO	8.53387E+07	3.25715E+08	8.15260E+03	1.13940E+04	4.70721E+02	2.48859E+01
DE	**3.00392E+03**	**4.05894E+03**	2.08036E+04	4.89037E+03	4.86317E+02	2.04412E+00
PSO	2.03442E+08	2.57266E+08	6.63641E+02	4.47314E+01	5.05592E+02	2.15040E+01
MVO	9.36354E+03	5.53919E+03	3.00383E+02	1.53520E-01	4.86372E+02	4.95954E+00
PO	7.02543E+07	9.55892E+07	4.90161E+03	2.67373E+03	5.09506E+02	2.24434E+01
RIME	8.74199E+03	6.48683E+03	3.01617E+02	5.70317E-01	4.83596E+02	2.83902E+01
HHO	1.21041E+07	2.26252E+06	7.93864E+03	3.11025E+03	5.28546E+02	3.11639E+01
KSO	2.35274E+05	7.02423E+04	1.86133E+03	8.00625E+02	4.91172E+02	3.24230E+00
SCA	1.23422E+10	2.32735E+09	3.72628E+04	7.71070E+03	1.51572E+03	2.39636E+02
	F4		F5		F6	
	Avg	Std	Avg	Std	Avg	Std
IPCACO	5.52969E+02	1.32655E+01	6.00029E+02	6.54552E-02	7.84835E+02	1.54533E+01
ACO	6.71104E+02	3.14020E+01	6.00883E+02	1.32201E+00	9.16971E+02	1.97209E+01
DE	6.06310E+02	1.07347E+01	**6.00000E+02**	**0.00000E+00**	8.43092E+02	9.74752E+00
PSO	7.44759E+02	2.88843E+01	6.51909E+02	1.06378E+01	9.20732E+02	1.63008E+01
MVO	5.85958E+02	2.63518E+01	6.11070E+02	8.18309E+00	8.28019E+02	2.30854E+01
PO	7.28951E+02	4.57355E+01	6.58206E+02	8.99638E+00	1.12828E+03	6.82664E+01
RIME	5.80606E+02	2.26456E+01	6.00313E+02	2.28856E-01	8.17758E+02	2.14418E+01
HHO	7.45115E+02	3.22300E+01	6.59595E+02	7.81539E+00	1.22945E+03	8.14883E+01
KSO	**5.50740E+02**	**1.29537E+01**	6.01416E+02	3.40845E-01	**7.69739E+02**	**1.04589E+01**
SCA	7.73721E+02	1.88100E+01	6.48169E+02	4.55944E+00	1.12872E+03	3.21097E+01
	F7		F8		F9	
	Avg	Std	Avg	Std	Avg	Std
IPCACO	8.55458E+02	1.73591E+01	9.32673E+02	3.85160E+01	4.30303E+03	7.19766E+02
ACO	9.60522E+02	5.10079E+01	1.21663E+03	6.66491E+02	5.11891E+03	1.96770E+03
DE	9.05586E+02	9.75910E+00	**9.00000E+02**	**1.03423E-13**	5.92373E+03	2.12196E+02
PSO	1.00071E+03	3.18126E+01	5.31698E+03	3.17545E+03	5.96325E+03	5.86578E+02
MVO	8.94132E+02	2.56494E+01	2.34869E+03	2.44256E+03	4.07483E+03	6.14979E+02
PO	9.79864E+02	3.46146E+01	5.19975E+03	6.66819E+02	5.74018E+03	7.29228E+02
RIME	8.72248E+02	1.63691E+01	1.26193E+03	3.72859E+02	3.77354E+03	4.88833E+02
HHO	9.72883E+02	2.02031E+01	6.79390E+03	8.21069E+02	5.32807E+03	4.90870E+02
KSO	**8.54067E+02**	**1.31346E+01**	9.13736E+02	1.13685E+01	**3.48776E+03**	**5.75851E+02**
SCA	1.04342E+03	1.93419E+01	5.15591E+03	8.62862E+02	8.09357E+03	4.04687E+02
	F10		F11		F12	
	Avg	Std	Avg	Std	Avg	Std
IPCACO	**1.14358E+03**	**2.82290E+01**	6.29102E+04	4.08813E+04	1.74813E+04	1.79448E+04
ACO	1.24909E+03	5.86129E+01	**5.79433E+04**	**7.12628E+04**	1.87556E+04	1.85946E+04
DE	1.16221E+03	2.40794E+01	1.73896E+06	9.14549E+05	3.05190E+04	1.40452E+04
PSO	1.29075E+03	4.05331E+01	2.62774E+07	1.15954E+07	4.82941E+06	1.36667E+06
MVO	1.27077E+03	6.52741E+01	4.25987E+06	3.22471E+06	7.54744E+04	4.49998E+04
PO	1.28827E+03	7.82673E+01	2.72009E+07	2.86553E+07	1.55611E+05	1.51894E+05
RIME	1.18736E+03	3.62528E+01	1.75824E+06	1.36391E+06	2.17579E+04	1.91467E+04
HHO	1.24433E+03	4.18191E+01	1.18959E+07	7.55480E+06	4.01671E+05	2.26845E+05

KSO	1.17318E+03	3.04754E+01	1.73620E+06	5.85321E+05	**9.72809E+03**	**7.92685E+03**
SCA	2.18285E+03	4.55413E+02	1.20629E+09	2.88371E+08	4.33002E+08	1.45149E+08
	F13		F14		F15	
	Avg	Std	Avg	Std	Avg	Std
IPCACO	**6.12471E+03**	**5.31557E+03**	**3.81704E+03**	**3.37804E+03**	2.19481E+03	2.40426E+02
ACO	3.69276E+05	1.91404E+06	1.56027E+04	1.40505E+04	2.30780E+03	3.85217E+02
DE	5.07647E+04	3.35966E+04	8.96565E+03	6.05363E+03	**2.06343E+03**	**1.38751E+02**
PSO	1.25996E+04	2.19929E+04	3.83874E+05	1.89262E+05	2.93474E+03	2.55166E+02
MVO	6.78647E+03	4.61952E+03	1.58316E+04	1.40857E+04	2.38798E+03	2.70502E+02
PO	5.80191E+04	7.66612E+04	6.95460E+04	4.74629E+04	3.12103E+03	2.98593E+02
RIME	1.42917E+04	7.80456E+03	1.13407E+04	1.16600E+04	2.38776E+03	2.98776E+02
HHO	6.11063E+04	7.59745E+04	6.93783E+04	4.66362E+04	3.23941E+03	4.14178E+02
KSO	1.40077E+05	1.49175E+05	7.41724E+03	8.79389E+03	2.34453E+03	2.20287E+02
SCA	1.41650E+05	7.57373E+04	1.39736E+07	1.66471E+07	3.56842E+03	1.72449E+02
	F16		F17		F18	
	Avg	Std	Avg	Std	Avg	Std
IPCACO	1.90924E+03	1.33909E+02	**8.94544E+04**	**8.45784E+04**	9.18813E+03	9.91682E+03
ACO	2.09700E+03	3.15258E+02	4.58769E+05	4.98225E+05	1.56504E+04	1.64428E+04
DE	1.86899E+03	7.92272E+01	3.07340E+05	1.65898E+05	**8.88473E+03**	**5.96494E+03**
PSO	2.39099E+03	2.25275E+02	2.29463E+05	1.72033E+05	1.29877E+06	6.13693E+05
MVO	2.02590E+03	1.57055E+02	1.96010E+05	1.09019E+05	2.26007E+04	1.73823E+04
PO	2.37943E+03	2.20442E+02	5.84836E+05	5.47542E+05	6.19480E+05	4.08821E+05
RIME	2.06366E+03	2.11665E+02	2.44613E+05	2.37051E+05	1.73207E+04	1.54847E+04
HHO	2.56220E+03	2.89722E+02	1.08962E+06	1.20785E+06	3.59038E+05	2.05150E+05
KSO	**1.86283E+03**	**1.14168E+02**	1.80719E+06	1.58223E+06	9.80800E+03	8.34380E+03
SCA	2.42048E+03	1.57744E+02	3.06318E+06	1.60617E+06	2.54230E+07	1.29469E+07
	F19		F20		F21	
	Avg	Std	Avg	Std	Avg	Std
IPCACO	2.24554E+03	1.39932E+02	2.35470E+03	1.73531E+01	4.35894E+03	1.86270E+03
ACO	2.28040E+03	2.03389E+02	2.45459E+03	4.70128E+01	6.92069E+03	1.94572E+03
DE	**2.13193E+03**	**6.74182E+01**	2.40731E+03	1.14316E+01	3.53291E+03	1.57222E+03
PSO	2.55539E+03	1.53443E+02	2.53403E+03	3.86415E+01	5.56308E+03	2.70954E+03
MVO	2.37518E+03	1.39843E+02	2.38616E+03	2.04333E+01	4.80431E+03	1.51419E+03
PO	2.54154E+03	1.32310E+02	2.50168E+03	4.14994E+01	3.19832E+03	1.64646E+03
RIME	2.30559E+03	1.77209E+02	2.38565E+03	2.11798E+01	4.25380E+03	1.37112E+03
HHO	2.75491E+03	2.44810E+02	2.55169E+03	4.01526E+01	6.29967E+03	1.90510E+03
KSO	2.27193E+03	1.58026E+02	**2.35292E+03**	**1.55991E+01**	**2.74766E+03**	**1.15764E+03**
SCA	2.60123E+03	1.30927E+02	2.55102E+03	2.27281E+01	7.41539E+03	2.85095E+03
	F22		F23		F24	
	Avg	Std	Avg	Std	Avg	Std
IPCACO	2.70410E+03	1.78220E+01	**2.86810E+03**	**1.36618E+01**	2.89458E+03	1.52467E+01
ACO	2.75497E+03	5.71237E+01	3.00091E+03	3.52359E+01	2.89270E+03	1.77316E+01
DE	2.75965E+03	9.08267E+00	2.95972E+03	1.34678E+01	2.88739E+03	3.22824E-01
PSO	3.13292E+03	1.27584E+02	3.19216E+03	9.80796E+01	2.90927E+03	1.94734E+01
MVO	2.73489E+03	2.67441E+01	2.89733E+03	2.58274E+01	**2.88691E+03**	**8.17121E-02**
PO	2.95503E+03	6.83709E+01	3.10167E+03	6.85425E+01	2.93259E+03	3.26995E+01
RIME	2.73274E+03	1.81170E+01	2.92939E+03	3.10959E+01	2.89366E+03	1.11269E+01
HHO	3.15358E+03	9.06118E+01	3.45663E+03	1.75770E+02	2.90235E+03	1.57482E+01

KSO	**2.69373E+03**	**1.42323E+01**	2.87970E+03	1.82778E+01	2.88890E+03	5.00868E-01
SCA	2.98185E+03	2.69686E+01	3.16314E+03	3.57276E+01	3.22573E+03	9.24319E+01

	F25		F26		F27	
	Avg	Std	Avg	Std	Avg	Std
IPCACO	4.06514E+03	5.97334E+02	3.22814E+03	1.30528E+01	**3.11397E+03**	**3.63004E+01**
ACO	4.63092E+03	5.37154E+02	3.22709E+03	1.35361E+01	3.22683E+03	4.56500E+01
DE	4.64831E+03	9.59717E+01	**3.20616E+03**	**3.51947E+00**	3.20121E+03	3.87591E+01
PSO	5.09186E+03	2.06588E+03	3.39913E+03	1.22436E+02	3.24318E+03	2.07742E+01
MVO	4.66486E+03	3.29138E+02	3.21638E+03	1.84790E+01	3.21236E+03	5.41149E+01
PO	6.39319E+03	1.55635E+03	3.31582E+03	5.16447E+01	3.30237E+03	3.56428E+01
RIME	4.36214E+03	6.34883E+02	3.22479E+03	1.03453E+01	3.22698E+03	2.87569E+01
HHO	6.62439E+03	1.79135E+03	3.34723E+03	7.06743E+01	3.25998E+03	2.38092E+01
KSO	**4.05290E+03**	**1.51687E+02**	3.22573E+03	1.37383E+01	3.22655E+03	4.34467E+00
SCA	6.96955E+03	2.89248E+02	3.40902E+03	4.56283E+01	3.78165E+03	1.21809E+02

	F28		F29	
	Avg	Std	Avg	Std
IPCACO	3.56072E+03	1.86102E+02	**8.07918E+03**	**1.92094E+03**
ACO	3.62635E+03	1.97519E+02	2.35927E+04	5.56996E+04
DE	**3.50533E+03**	**7.29328E+01**	1.35498E+04	5.11128E+03
PSO	4.31883E+03	2.56050E+02	3.34557E+06	1.41752E+06
MVO	3.74475E+03	1.82535E+02	7.15862E+05	6.96855E+05
PO	4.55895E+03	3.86382E+02	6.63087E+06	5.64255E+06
RIME	3.73131E+03	1.35206E+02	1.58759E+04	1.21160E+04
HHO	4.40472E+03	3.34552E+02	1.86764E+06	9.79238E+05
KSO	3.53701E+03	1.32121E+02	4.31884E+04	1.42750E+04
SCA	4.64037E+03	2.47403E+02	7.60412E+07	2.77206E+07

Table S2. P-value obtained from WST on IPCACO and comparative algorithms.

	ACO	DE	PSO	MVO	PO	RIME	HHO	KSO	SCA
F1	2.95746E-03	5.03833E-01	1.73440E-06	6.89229E-05	1.73440E-06	2.83079E-04	1.73440E-06	1.73440E-06	1.73440E-06
F2	1.73440E-06	1.73440E-06	1.73440E-06	1.73440E-06	1.73440E-06	1.73440E-06	1.73440E-06	1.73440E-06	1.73440E-06
F3	3.06500E-04	1.73440E-06	2.12664E-06	1.73440E-06	1.73440E-06	7.51366E-05	1.73440E-06	1.73440E-06	1.73440E-06
F4	1.73440E-06	1.73440E-06	1.73440E-06	6.98378E-06	1.73440E-06	1.49356E-05	1.73440E-06	7.34325E-01	1.73440E-06
F5	1.14992E-04	1.73440E-06	1.73440E-06	1.73440E-06	1.73440E-06	1.92092E-06	1.73440E-06	1.73440E-06	1.73440E-06
F6	1.73440E-06	1.73440E-06	1.73440E-06	1.73440E-06	1.73440E-06	1.63945E-05	1.73440E-06	6.63921E-04	1.73440E-06
F7	3.18168E-06	1.73440E-06	1.73440E-06	2.35342E-06	1.73440E-06	3.06500E-04	1.73440E-06	7.49871E-01	1.73440E-06
F8	1.75184E-02	1.73440E-06	1.73440E-06	1.14992E-04	1.73440E-06	9.31566E-06	1.73440E-06	1.85190E-02	1.73440E-06
F9	4.07023E-02	1.92092E-06	3.51524E-06	2.36936E-01	1.73440E-06	1.03568E-03	1.97295E-05	1.47728E-04	1.73440E-06
F10	2.35342E-06	6.83586E-03	1.73440E-06	1.92092E-06	1.73440E-06	2.05153E-04	1.73440E-06	3.31726E-04	1.73440E-06
F11	4.94980E-02	1.73440E-06	1.73440E-06	1.73440E-06	1.73440E-06	1.92092E-06	1.73440E-06	1.73440E-06	1.73440E-06
F12	6.28843E-01	5.66717E-03	1.73440E-06	6.98378E-06	4.72920E-06	4.65283E-01	1.73440E-06	1.65027E-01	1.73440E-06
F13	1.63945E-05	1.73440E-06	9.27103E-03	4.77947E-01	2.35342E-06	3.88111E-04	3.18168E-06	1.92092E-06	1.73440E-06
F14	1.89097E-04	3.88111E-04	1.73440E-06	8.18775E-05	1.73440E-06	2.58456E-03	1.73440E-06	5.98356E-02	1.73440E-06
F15	1.84622E-01	2.30381E-02	1.73440E-06	1.31942E-02	1.92092E-06	2.06711E-02	1.92092E-06	1.75184E-02	1.73440E-06
F16	2.76527E-03	2.62299E-01	1.92092E-06	2.76527E-03	1.73440E-06	2.25512E-03	1.73440E-06	1.98610E-01	1.73440E-06
F17	2.22483E-04	1.12654E-05	9.71105E-05	4.89690E-04	1.02463E-05	1.28663E-03	5.21649E-06	1.73440E-06	1.73440E-06
F18	5.70965E-02	7.03564E-01	1.73440E-06	5.70644E-04	1.73440E-06	5.70965E-02	1.73440E-06	6.14315E-01	1.73440E-06
F19	4.90798E-01	1.11380E-03	2.87860E-06	9.27103E-03	1.73440E-06	1.77907E-01	1.92092E-06	4.90798E-01	1.73440E-06

F20	2.87860E-06	1.92092E-06	1.73440E-06	2.59671E-05	1.73440E-06	4.86026E-05	1.73440E-06	4.04835E-01	1.73440E-06
F21	1.05695E-04	8.97178E-02	6.26828E-02	3.70935E-01	1.02011E-01	8.77403E-01	9.71105E-05	6.83586E-03	9.71105E-05
F22	3.31726E-04	1.73440E-06	1.73440E-06	1.14992E-04	1.73440E-06	4.44934E-05	1.73440E-06	1.47954E-02	1.73440E-06
F23	1.92092E-06	1.73440E-06	1.73440E-06	2.37045E-05	1.73440E-06	2.35342E-06	1.73440E-06	6.83586E-03	1.73440E-06
F24	3.93334E-01	8.21674E-03	1.19734E-03	1.96458E-03	1.02463E-05	6.28843E-01	2.56371E-02	8.61213E-01	1.73440E-06
F25	1.19734E-03	8.46608E-06	9.27103E-03	8.91873E-05	8.46608E-06	3.87230E-02	7.69086E-06	2.28880E-01	1.73440E-06
F26	6.14315E-01	1.73440E-06	1.73440E-06	5.66717E-03	1.73440E-06	2.89477E-01	1.73440E-06	4.16534E-01	1.73440E-06
F27	2.84510E-06	3.90166E-06	1.73440E-06	1.73440E-06	1.73440E-06	2.35342E-06	1.73440E-06	1.73440E-06	1.73440E-06
F28	1.30592E-01	2.36936E-01	1.73440E-06	1.96458E-03	1.73440E-06	1.28663E-03	1.73440E-06	7.34325E-01	1.73440E-06
F29	4.53356E-04	3.11232E-05	1.73440E-06	1.73440E-06	1.73440E-06	1.97295E-05	1.73440E-06	1.73440E-06	1.73440E-06

Table S3. Results of IPCACO and SOTA algorithms.

	F1		F2		F3	
	Avg	Std	Avg	Std	Avg	Std
IPCACO	4.11478E+03	3.99154E+03	3.00000E+02	1.57073E-09	4.50895E+02	3.33353E+01
MALBFOA	2.22441E+03	3.43717E+03	3.50291E+02	1.04154E+02	4.55249E+02	2.84730E+01
ASCA-PSO	1.93224E+08	3.09603E+08	1.28982E+03	3.54052E+02	5.22571E+02	3.33002E+01
HG_SMA	3.08093E+08	2.06356E+08	1.58348E+04	4.86910E+03	5.12433E+02	2.69703E+01
FSTPSO	1.86809E+10	6.15827E+09	8.90289E+04	2.55979E+04	3.61858E+03	1.46782E+03
CGSCA	1.33914E+10	2.33067E+09	4.17665E+04	5.68681E+03	1.71937E+03	2.72798E+02
CGPSO	1.37927E+08	1.63374E+07	8.64524E+02	8.18612E+01	4.85391E+02	2.55865E+01
SCDWOA	**1.00000E+02**	**1.42230E-04**	**3.00000E+02**	**5.37609E-11**	**4.19216E+02**	**2.15296E+01**
ISMA	4.45458E+08	9.57297E+08	1.09996E+04	7.06986E+03	5.10779E+02	3.04851E+01
WEMFO	1.13333E+04	7.96074E+03	9.86123E+03	4.24049E+03	5.00286E+02	5.55083E+01
ALGSA	2.81297E+10	8.13839E+09	1.24095E+05	5.86979E+04	1.07508E+03	4.29486E+02
SRWPSO	1.57112E+03	1.70363E+03	3.00000E+02	3.03772E-05	4.54692E+02	2.57847E+01
	F4		F5		F6	
	Avg	Std	Avg	Std	Avg	Std
IPCACO	**5.49389E+02**	**1.77726E+01**	**6.00031E+02**	**7.84821E-02**	**7.82530E+02**	**1.70168E+01**
MALBFOA	7.06185E+02	3.72555E+01	6.38740E+02	5.30692E+00	9.46337E+02	3.39733E+01
ASCA-PSO	7.27002E+02	4.20167E+01	6.30510E+02	1.14589E+01	9.83685E+02	3.12968E+01
HG_SMA	6.55255E+02	2.85439E+01	6.30093E+02	8.34981E+00	9.63091E+02	2.90812E+01
FSTPSO	8.07045E+02	3.51274E+01	6.65682E+02	8.40600E+00	1.32230E+03	1.08798E+02
CGSCA	7.95478E+02	2.03023E+01	6.55513E+02	6.20055E+00	1.14571E+03	3.48019E+01
CGPSO	7.67050E+02	3.69194E+01	6.54473E+02	1.06201E+01	9.34362E+02	2.07703E+01
SCDWOA	7.10134E+02	6.07425E+01	6.39874E+02	1.09322E+01	1.07681E+03	7.58051E+01
ISMA	6.31251E+02	2.46791E+01	6.16499E+02	7.30472E+00	9.14487E+02	4.33556E+01
WEMFO	6.72651E+02	4.13008E+01	6.31068E+02	1.21218E+01	9.32700E+02	7.41987E+01
ALGSA	7.15980E+02	4.76031E+01	6.61929E+02	1.08413E+01	1.77248E+03	2.57999E+02
SRWPSO	6.14718E+02	2.70822E+01	6.18899E+02	5.23424E+00	8.79960E+02	4.45617E+01
	F7		F8		F9	
	Avg	Std	Avg	Std	Avg	Std
IPCACO	**8.56381E+02**	**1.67394E+01**	**9.23151E+02**	**2.57156E+01**	4.21684E+03	6.25170E+02
MALBFOA	1.00114E+03	2.43365E+01	7.44062E+03	1.21207E+03	4.06805E+03	4.08833E+02
ASCA-PSO	9.97208E+02	2.90135E+01	4.57401E+03	2.01221E+03	6.19315E+03	1.13666E+03
HG_SMA	9.46629E+02	3.21110E+01	4.56192E+03	1.70298E+03	5.27372E+03	7.40306E+02
FSTPSO	1.04979E+03	3.47538E+01	7.00874E+03	2.10792E+03	6.88917E+03	6.41258E+02
CGSCA	1.05702E+03	1.95898E+01	6.28670E+03	1.05288E+03	8.07464E+03	3.09145E+02
CGPSO	1.01095E+03	2.30587E+01	5.91601E+03	1.97934E+03	6.17973E+03	5.55393E+02
SCDWOA	9.59723E+02	4.05572E+01	3.90242E+03	1.24873E+03	4.72882E+03	5.01794E+02
ISMA	9.16886E+02	2.71150E+01	3.45581E+03	9.78960E+02	4.77809E+03	7.65832E+02
WEMFO	9.87997E+02	3.65645E+01	5.95386E+03	2.48905E+03	5.28190E+03	7.56317E+02
ALGSA	1.01890E+03	4.10169E+01	6.71099E+03	3.00070E+03	7.38235E+03	5.24577E+02
SRWPSO	8.78933E+02	2.15911E+01	2.52455E+03	5.11105E+02	**3.73559E+03**	**5.24948E+02**
	F10		F11		F12	
	Avg	Std	Avg	Std	Avg	Std
IPCACO	**1.13595E+03**	**2.71398E+01**	6.51300E+04	3.67337E+04	2.39505E+04	1.74441E+04

MALBFOA	1.20008E+03	3.00060E+01	4.62055E+05	2.96645E+05	4.76178E+04	1.69230E+04
ASCA-PSO	1.30663E+03	4.86828E+01	6.56801E+07	1.08561E+08	8.39144E+06	2.60925E+06
HG_SMA	1.32457E+03	6.07383E+01	3.85549E+07	2.71105E+07	9.91206E+05	2.15052E+06
FSTPSO	4.09025E+03	1.75858E+03	1.69618E+09	9.46376E+08	4.64050E+08	9.26493E+08
CGSCA	2.23263E+03	2.74463E+02	1.40658E+09	3.17597E+08	4.60760E+08	1.37782E+08
CGPSO	1.28717E+03	3.16932E+01	2.68131E+07	1.00864E+07	4.58927E+06	1.37711E+06
SCDWOA	1.22868E+03	4.42277E+01	4.70297E+04	8.01528E+04	**1.81762E+04**	**1.63178E+04**
ISMA	1.24479E+03	5.97633E+01	3.33978E+06	4.45915E+06	2.73671E+05	1.33134E+06
WEMFO	1.35777E+03	9.29579E+01	1.86507E+06	1.38635E+06	9.46625E+04	1.29306E+05
ALGSA	7.93482E+03	9.93420E+03	1.74416E+09	1.52295E+09	2.11246E+08	4.84105E+08
SRWPSO	1.20858E+03	3.13303E+01	**3.55071E+04**	**3.79710E+04**	1.66112E+05	8.07903E+05
	F13		F14		F15	
	Avg	Std	Avg	Std	Avg	Std
IPCACO	8.92564E+03	1.18483E+04	6.34876E+03	5.44978E+03	**2.23048E+03**	**2.59450E+02**
MALBFOA	3.93628E+03	2.72012E+03	2.05875E+04	7.98499E+03	2.57349E+03	2.24031E+02
ASCA-PSO	2.96958E+04	1.81716E+04	1.29418E+06	3.57730E+05	2.92036E+03	2.91143E+02
HG_SMA	5.91008E+04	4.96010E+04	8.53004E+04	7.62472E+04	2.74658E+03	3.64742E+02
FSTPSO	8.83662E+04	9.27288E+04	3.50648E+04	2.16598E+04	3.80122E+03	4.12531E+02
CGSCA	1.64123E+05	8.23486E+04	9.83161E+06	1.21631E+07	3.63914E+03	2.22697E+02
CGPSO	1.22094E+04	9.29083E+03	5.34824E+05	2.35262E+05	2.91454E+03	2.90965E+02
SCDWOA	**1.60349E+03**	**6.71044E+01**	**3.26923E+03**	**4.73542E+03**	2.55047E+03	3.56779E+02
ISMA	5.19584E+04	5.42775E+04	7.01961E+03	3.31292E+03	2.63311E+03	3.89763E+02
WEMFO	5.57017E+04	5.14815E+04	5.71649E+04	4.03506E+04	2.70588E+03	2.90722E+02
ALGSA	8.00769E+05	2.32315E+06	3.45010E+04	6.29974E+03	2.90040E+03	3.97072E+02
SRWPSO	7.41634E+03	4.71760E+03	5.97010E+03	5.50202E+03	2.49033E+03	2.55220E+02
	F16		F17		F18	
	Avg	Std	Avg	Std	Avg	Std
IPCACO	**1.95498E+03**	**1.80289E+02**	7.70343E+04	7.02170E+04	6.39384E+03	4.59311E+03
MALBFOA	2.21389E+03	1.34651E+02	9.16948E+04	3.40744E+04	1.07721E+04	4.88121E+03
ASCA-PSO	2.25356E+03	1.93721E+02	4.60610E+05	3.75739E+05	3.68450E+06	2.36436E+06
HG_SMA	2.14281E+03	1.68057E+02	8.21309E+05	6.03206E+05	4.16214E+05	3.39345E+05
FSTPSO	2.63975E+03	3.32861E+02	1.78157E+06	2.18368E+06	3.38967E+06	3.27254E+06
CGSCA	2.51352E+03	1.55648E+02	4.01335E+06	2.49403E+06	2.40082E+07	1.07526E+07
CGPSO	2.25253E+03	1.71405E+02	2.14879E+05	8.58295E+04	1.55286E+06	6.25695E+05
SCDWOA	2.34033E+03	2.69618E+02	**1.11349E+04**	**1.18098E+04**	**3.80167E+03**	**9.75280E+03**
ISMA	2.15742E+03	2.23402E+02	4.46393E+05	3.87984E+05	6.92488E+03	6.61277E+03
WEMFO	2.22002E+03	2.14539E+02	6.66675E+05	4.46860E+05	4.00816E+04	5.17520E+04
ALGSA	2.03930E+03	2.36690E+02	1.44471E+06	4.05780E+06	2.41097E+07	5.57248E+07
SRWPSO	2.23070E+03	2.39469E+02	8.51943E+04	6.36132E+04	8.56634E+03	5.84802E+03
	F19		F20		F21	
	Avg	Std	Avg	Std	Avg	Std
IPCACO	**2.25776E+03**	**1.11419E+02**	**2.34709E+03**	**1.43442E+01**	3.61563E+03	1.78960E+03
MALBFOA	2.53958E+03	1.63917E+02	2.50295E+03	2.87378E+01	3.49739E+03	1.62074E+03
ASCA-PSO	2.50880E+03	1.69825E+02	2.48944E+03	2.67188E+01	6.31074E+03	2.13159E+03
HG_SMA	2.41330E+03	1.33140E+02	2.43311E+03	2.87072E+01	**2.39950E+03**	**3.13574E+01**
FSTPSO	2.85962E+03	1.94403E+02	2.60468E+03	4.86419E+01	6.80597E+03	1.60276E+03
CGSCA	2.63575E+03	1.35644E+02	2.57004E+03	2.16538E+01	3.88911E+03	2.68490E+02
CGPSO	2.63459E+03	1.40463E+02	2.51462E+03	2.38805E+01	3.88257E+03	2.38681E+03
SCDWOA	2.58305E+03	2.22311E+02	2.47644E+03	3.90504E+01	5.38455E+03	1.95837E+03
ISMA	2.39825E+03	1.72460E+02	2.40417E+03	5.02204E+01	2.47547E+03	9.24366E+02
WEMFO	2.49727E+03	1.89975E+02	2.47640E+03	4.10838E+01	6.23276E+03	1.72382E+03
ALGSA	3.01980E+03	1.33899E+02	2.48621E+03	4.47765E+01	9.13188E+03	4.54993E+02
SRWPSO	2.42033E+03	1.37915E+02	2.39280E+03	1.59459E+01	2.53074E+03	8.76971E+02
	F22		F23		F24	
	Avg	Std	Avg	Std	Avg	Std
IPCACO	**2.70174E+03**	**1.43874E+01**	**2.86995E+03**	**1.56671E+01**	2.89363E+03	1.22433E+01
MALBFOA	2.90613E+03	4.47536E+01	3.25095E+03	6.15662E+01	2.88583E+03	1.70149E+00
ASCA-PSO	2.86948E+03	4.52872E+01	3.02994E+03	3.49517E+01	2.92367E+03	3.17495E+01
HG_SMA	2.79260E+03	3.31150E+01	2.95200E+03	3.23583E+01	2.94104E+03	3.69237E+01
FSTPSO	3.33906E+03	1.40438E+02	3.41623E+03	1.30909E+02	3.83026E+03	2.89387E+02

CGSCA	2.99790E+03	2.71413E+01	3.15647E+03	2.57651E+01	3.26864E+03	6.38899E+01
CGPSO	3.07746E+03	9.27274E+01	3.17261E+03	8.05122E+01	2.92154E+03	2.55741E+01
SCDWOA	2.88170E+03	4.99097E+01	3.03131E+03	6.79580E+01	**2.88457E+03**	**1.81150E+01**
ISMA	2.76477E+03	3.19836E+01	2.93161E+03	3.47007E+01	2.90405E+03	2.15868E+01
WEMFO	2.82004E+03	3.75516E+01	2.96698E+03	3.81640E+01	2.89476E+03	1.67407E+01
ALGSA	2.88060E+03	3.96256E+01	3.04999E+03	2.72242E+01	3.97493E+03	7.37362E+02
SRWPSO	2.81033E+03	4.26082E+01	2.98207E+03	3.84829E+01	2.90068E+03	1.84872E+01

	F25		F26		F27	
	Avg	Std	Avg	Std	Avg	Std
IPCACO	**4.07946E+03**	**6.81001E+02**	3.23078E+03	1.07310E+01	3.15119E+03	6.56372E+01
MALBFOA	4.84710E+03	1.68500E+03	3.24591E+03	1.56228E+01	**3.13518E+03**	**4.10014E+01**
ASCA-PSO	6.34175E+03	7.76496E+02	3.29625E+03	3.56934E+01	3.29653E+03	7.19514E+01
HG_SMA	4.75262E+03	7.12583E+02	**3.20001E+03**	**2.82498E-04**	3.29659E+03	5.30504E+00
FSTPSO	9.17353E+03	1.31408E+03	3.75287E+03	2.95297E+02	4.65142E+03	4.80510E+02
CGSCA	7.18495E+03	3.78719E+02	3.39458E+03	4.99567E+01	3.92207E+03	1.55352E+02
CGPSO	5.84972E+03	1.82288E+03	3.21169E+03	8.28600E+01	3.26039E+03	2.44124E+01
SCDWOA	6.33320E+03	7.17287E+02	3.20001E+03	1.58005E-04	3.23453E+03	8.64859E+01
ISMA	4.40549E+03	8.63672E+02	3.20001E+03	2.11489E-04	3.29949E+03	1.98656E+00
WEMFO	5.63326E+03	5.14330E+02	3.24350E+03	2.89945E+01	3.25371E+03	4.47105E+01
ALGSA	5.82307E+03	6.56302E+02	3.31687E+03	3.48646E+01	5.65688E+03	7.06016E+02
SRWPSO	5.07263E+03	1.62131E+03	3.28199E+03	3.50859E+01	3.19941E+03	4.69834E+01

	F28		F29	
	Avg	Std	Avg	Std
IPCACO	**3.53172E+03**	**1.72300E+02**	8.29513E+03	2.89867E+03
MALBFOA	3.77832E+03	1.55187E+02	6.93384E+04	2.51709E+04
ASCA-PSO	4.35150E+03	1.94514E+02	8.75655E+06	5.80832E+06
HG_SMA	3.85193E+03	2.24114E+02	3.48710E+06	3.30217E+06
FSTPSO	5.67231E+03	6.73772E+02	2.69952E+07	2.76622E+07
CGSCA	4.74963E+03	2.38152E+02	8.77100E+07	3.34442E+07
CGPSO	4.31616E+03	3.09556E+02	4.70973E+06	1.88252E+06
SCDWOA	3.82285E+03	3.64684E+02	**6.11784E+03**	**4.14240E+03**
ISMA	3.90284E+03	2.95905E+02	2.28462E+05	3.29022E+05
WEMFO	4.06138E+03	2.91097E+02	5.95120E+05	1.37302E+06
ALGSA	3.80646E+03	2.67968E+02	2.09124E+07	9.41645E+07
SRWPSO	3.94051E+03	1.65268E+02	8.90308E+03	2.51770E+03

Table S4. P-value obtained from WSRT on IPCACO and SOTA algorithms.

	MALBFOA	ASCA-PSO	HG_SMA	FSTPSO	CGSCA	CGPSO	SCDWOA	ISMA	WEMFO	ALGSA	SRWPSO
F1	7.19033E-02	1.73440E-06	1.73440E-06	1.73440E-06	1.73440E-06	1.73440E-06	1.73440E-06	4.19551E-04	2.83079E-04	1.73440E-06	7.73094E-03
F2	1.73440E-06	1.73440E-06	1.73440E-06	1.73440E-06	1.73440E-06	1.73440E-06	1.49519E-02	1.73440E-06	1.73440E-06	1.73440E-06	5.21649E-06
F3	4.16534E-01	1.92092E-06	5.21649E-06	1.73440E-06	1.73440E-06	5.70644E-04	2.10526E-03	1.02463E-05	1.35948E-04	1.73440E-06	6.28843E-01
F4	1.73440E-06	1.73440E-06	1.73440E-06	1.73440E-06	1.73440E-06	1.73440E-06	1.73440E-06	1.73440E-06	1.73440E-06	1.73440E-06	2.35342E-06
F5	1.73440E-06	1.73440E-06	1.73440E-06	1.73440E-06	1.73440E-06	1.73440E-06	1.73440E-06	1.73440E-06	1.73440E-06	1.73440E-06	1.73440E-06
F6	1.73440E-06	1.73440E-06	1.73440E-06	1.73440E-06	1.73440E-06	1.73440E-06	1.73440E-06	1.73440E-06	1.73440E-06	1.73440E-06	1.73440E-06
F7	1.73440E-06	1.73440E-06	1.73440E-06	1.73440E-06	1.73440E-06	1.73440E-06	1.73440E-06	1.92092E-06	1.73440E-06	1.73440E-06	1.47728E-04
F8	1.73440E-06	1.73440E-06	1.73440E-06	1.73440E-06	1.73440E-06	1.73440E-06	1.73440E-06	1.73440E-06	1.73440E-06	1.73440E-06	1.73440E-06
F9	3.49346E-01	1.92092E-06	1.63945E-05	1.73440E-06	1.73440E-06	1.73440E-06	1.96458E-03	6.03501E-03	2.84342E-05	1.73440E-06	3.37885E-03
F10	4.28569E-06	1.73440E-06	1.73440E-06	1.73440E-06	1.73440E-06	1.73440E-06	2.60333E-06	1.73440E-06	1.73440E-06	4.44934E-05	5.21649E-06
F11	1.73440E-06	1.73440E-06	1.73440E-06	1.73440E-06	1.73440E-06	1.73440E-06	9.62659E-04	1.73440E-06	1.73440E-06	1.73440E-06	3.31726E-04
F12	1.89097E-04	1.73440E-06	1.73440E-06	1.73440E-06	1.73440E-06	1.73440E-06	1.35908E-01	3.08615E-01	1.97295E-05	2.83079E-04	5.17048E-01
F13	1.17481E-02	8.18775E-05	7.69086E-06	7.51366E-05	1.73440E-06	1.31942E-02	1.73440E-06	7.69086E-06	2.37045E-05	2.84342E-05	6.28843E-01
F14	7.69086E-06	1.73440E-06	1.73440E-06	2.35342E-06	1.73440E-06	1.73440E-06	5.31968E-03	3.60039E-01	1.73440E-06	1.73440E-06	5.99936E-01
F15	5.79245E-05	1.73440E-06	1.36011E-05	1.73440E-06	1.73440E-06	2.12664E-06	1.03568E-03	1.47728E-04	2.37045E-05	4.28569E-06	5.28725E-04
F16	8.46608E-06	1.79885E-05	3.88111E-04	1.92092E-06	2.12664E-06	1.49356E-05	8.46608E-06	1.19734E-03	4.53356E-04	8.58958E-02	3.88111E-04
F17	2.62299E-01	1.92092E-06	1.73440E-06	3.88218E-06	1.73440E-06	2.84342E-05	6.33914E-06	1.97295E-05	2.35342E-06	3.51524E-06	4.90798E-01

F18	1.19734E-03	1.73440E-06	2.12664E-06	1.73440E-06	1.73440E-06	1.73440E-06	6.89229E-05	9.58990E-01	3.85424E-03	2.12664E-06	9.36756E-02
F19	1.36011E-05	2.84342E-05	1.89097E-04	1.73440E-06	1.92092E-06	1.92092E-06	7.69086E-06	7.51366E-05	1.12654E-05	1.73440E-06	1.60464E-04
F20	1.73440E-06	1.73440E-06	1.73440E-06	1.73440E-06	1.73440E-06	1.73440E-06	1.73440E-06	2.59671E-05	1.73440E-06	1.73440E-06	1.92092E-06
F21	9.42611E-01	2.84342E-05	3.82034E-01	9.31566E-06	5.57743E-01	1.10926E-01	1.11380E-03	4.52807E-01	1.35940E-05	1.73440E-06	4.95525E-02
F22	1.73440E-06	1.73440E-06	1.73440E-06	1.73440E-06	1.73440E-06	1.73440E-06	1.73440E-06	1.73440E-06	1.73440E-06	1.73440E-06	1.73440E-06
F23	1.73440E-06	1.73440E-06	1.73440E-06	1.73440E-06	1.73440E-06	1.73440E-06	1.73440E-06	2.35342E-06	1.73440E-06	1.73440E-06	1.73440E-06
F24	9.71105E-05	5.79245E-05	1.73440E-06	1.73440E-06	1.73440E-06	1.89097E-04	3.58884E-04	3.68261E-02	7.49871E-01	1.73440E-06	5.70965E-02
F25	8.72967E-03	2.60333E-06	1.38204E-03	1.73440E-06	1.73440E-06	7.51366E-05	1.73440E-06	9.36756E-02	9.31566E-06	2.12664E-06	1.24526E-02
F26	8.94430E-04	1.73440E-06	1.73440E-06	1.73440E-06	1.73440E-06	2.56371E-02	1.73440E-06	1.73440E-06	1.85190E-02	1.73440E-06	2.60333E-06
F27	5.71646E-01	1.73440E-06	1.73440E-06	1.73440E-06	1.73440E-06	7.69086E-06	3.06500E-04	1.73440E-06	1.36011E-05	1.73440E-06	8.22358E-03
F28	1.60464E-04	1.73440E-06	1.23808E-05	1.73440E-06	1.73440E-06	1.92092E-06	6.89229E-05	1.63945E-05	1.73440E-06	1.79885E-05	3.51524E-06
F29	1.73440E-06	1.73440E-06	1.73440E-06	1.73440E-06	1.73440E-06	1.73440E-06	4.99155E-03	1.73440E-06	1.73440E-06	1.73440E-06	4.49189E-02

Table S5. The characteristics and underlying diseases of TSM and tuberculosis patients.

Variable	TSM(n=48)	pulmonary tuberculosis(n=96)	P
Age (year, mean±SD)	56.5±15.8	56.5±15.8	0.979
Gender, n (%)	/	/	1.000
male	40(83.3)	80(83.3)	/
female	8(16.7)	16(16.7)	/
Underlying diseases, n (%)	/	/	/
Hypertension	18(37.5)	31(32.3)	0.578
Diabetes mellitus	8(16.7)	24(25.0)	0.294
Solid organ tumors	7(14.6)	1(1.0)	0.003
Malignant tumor	7(14.6)	2(1.0)	0.011
Liver disease	11(22.9)	9(9.4)	0.027
Chronic renal insufficiency	14(29.2)	5(5.2)	0.000
Immune-related diseases	7(14.6)	0	0.001
Without Underlying diseases, n (%)	3(6.3)	37(38.5)	0.000

Notes: Immune-related diseases include anti-interferon-γ autoantibody-associated immunodeficiency (3), hyperimmunoglobulin E syndrome (2), systemic lupus erythematosus (1), and hemophagocytic syndrome (1).
